# Influence of Co and Mn Doping on the Surface Reconstruction of Faceted NiO(111) Nanosheets after the Oxygen Evolution Reaction

**DOI:** 10.1021/acs.jpcc.5c00493

**Published:** 2025-05-10

**Authors:** Konstantin K. Rücker, Dereje Hailu Taffa, Omeshwari Bisen, Marcel Risch, Darius Hayes, Elliot Brim, Ryan M. Richards, Corinna Harms, Michael Wark, Julian Lorenz

**Affiliations:** † Institute of Engineering Thermodynamics, 14930 German Aerospace Center (DLR), Carl-von-Ossietzky-Str.15, 26129 Oldenburg, Germany; ‡ Institute of Chemistry, Chemical Technology I, 11233 Carl von Ossietzky University of Oldenburg, Carl-von-Ossietzky-Str. 9-11, 26129 Oldenburg, Germany; § Nachwuchsgruppe Gestaltung des Sauerstoffentwicklungsmechanismus, 28340 Helmholtz-Zentrum Berlin für Materialien und Energie GmbH, Hahn-Meitner-Platz 1, 14109 Berlin, Germany; ∥ Department of Chemistry, 3557 Colorado School of Mines, 1500 Illinois Street, Golden, Colorado 80401, United States; ⊥ Chemical and Material Sciences Center, National Renewable Energy Laboratory, Golden, Colorado 80401, United States

## Abstract

Understanding dynamic surface reconstruction processes on transition metal oxides for the oxygen evolution reaction (OER) in alkaline electrolytes is crucial to the development of more active catalysts in water electrolysis technologies. Effective strategies in material development for activity enhancement include doping with additional transition metals and surface structuring through controlled exposure of defined surface facets. Here, a microwave-assisted synthesis route was used, that resulted in phase-pure Co- and Mn-doped NiO with various doping levels while maintaining the rock salt crystal structure of the pure, faceted NiO(111) nanosheets. X-ray diffraction and transmission electron microscopy showed an unaltered structure and morphology up to doping levels of 10 mol %. The impact of doping levels between 2 and 10% on the electrochemistry and OER overpotential was studied using the rotating disc electrode technique. A modest overpotential reduction of 34 mV was achieved for 5% Co-doping, being the most active material in the comparison, and an increase in overpotential of 56 mV for 10% Mn-doping, being the least active material, compared to the undoped NiO(111) material. Associated changes in the physical surface area and charges associated with surface redox reactions were aligned with detailed X-ray absorption spectroscopy and X-ray photoelectron spectroscopy analysis before and after electrochemical measurements, which showed different extents of surface reconstruction depending on the dopant and doping level. Thus, transformation of the less active rock salt structure to more active NiOOH functionalities was hampered by a low extent of surface reconstruction, explaining the modest activity enhancement after potentiodynamic cycling for 350 scans. The results demonstrate the effective synthesis of facet-controlled doped NiO-based model catalysts to scrutinize the impact of individual dopants on the electrochemical behavior and, thus the OER electrode activity.

## Graphical abstract

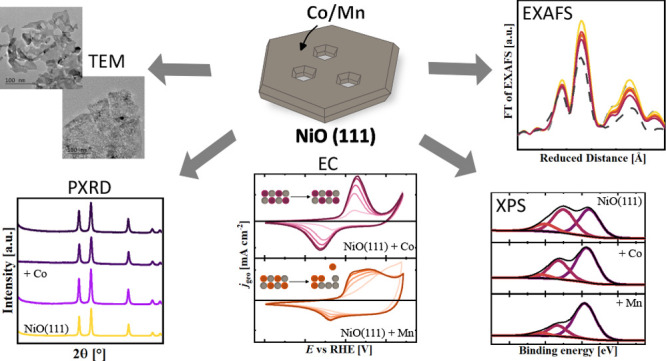

## 1 Introduction

The design of optimized electrocatalysts for electrochemical water electrolysis to produce hydrogen from renewable energy sources is one important step toward decarbonization of various industry sectors by substitution of fossil energy carriers.^1,2^ Water electrolysis allows the use of electricity from renewable energies to produce hydrogen via the hydrogen evolution reaction (HER), while oxygen evolves in the counter reaction via the oxygen evolution reaction (OER). The slow reaction kinetics of the OER, which exhibits a larger overpotential in comparison to the HER, results in a decrease in the overall energy efficiency of water electrolyzer technologies, requiring material development of the OER electrocatalysts.

In alkaline media, the OER can be catalyzed by platinum group metal (PGM)-free materials. Several transition metal oxides with different crystal structures, such as layer double hydroxides (LDH),^3,4^ spinel-type oxides,^5^ and rock-salt-type oxides,^6^ are considered efficient PGM-free OER electrocatalysts. Thereof, most active materials are based on mixtures of Ni, Co, Mn, and Fe rather than monometallic compounds.^7^ These catalysts transform during the OER, where surface reconstruction likely results in an amorphous surface layer of an NiOOH phase associated with redox changes of the incorporated transition metal centers.^8–12^ Thus, the as-synthesized catalyst is depicted as “pre-catalyst” that transforms dynamically into an active phase with a different structure during and back to the thermodynamically stable form in the sequence of OER electrocatalysis.

Material design strategies of transition metal-based OER catalysts resulted in advances in the synthesis and structural control to enhance the performance of these materials and provide an understanding of structure–property relationships. In general, activity enhancement is achieved by increasing the number of active sites, for example, via surface structuring and exposure of facets that are predominantly active for the OER, as well as by increasing the intrinsic activity using alloying or metal doping.^13,14^ For example, the synthesis of shape-controlled particles with a faceted surface enabled studies of well-defined catalyst systems.^15^ For a single-crystalline β-Co hydroxide, it was proven by multiple correlative *operando* microscopies that the OER mainly originated from the (1010)-edge facets of the corresponding nanosheets.^16^ Furthermore, it was suggested that ion (de)­intercalation happens faster on the edge positions.^16^ In accordance with the β-Co­(OH)_2_, NiO nanosheets display a multifold OER activity at the edges of the nanosheets compared to the basal planes.^16,17^ In a study on lanthanum nickelate perovskite thin films of different surface facets, it was concluded that the bulk facet orientation does influence the electrochemical activity after surface reconstruction.^18^ This was explained with a structural mismatch of the underlying (001) and (110) perovskite layer terminations to form the NiOOH active phase, whereas the (111) perovskite termination seems to favor the reconstruction to NiOOH, leading to lower OER overpotentials.^18^ Consequently, it is hypothesized that nanoparticulate faceted NiO will possess a comparable value as a well-defined system for investigating the impact of faceted precatalysts on the OER performance.

On the other hand, enhancing the intrinsic OER activity as well as the stability of Ni-based electrocatalysts by doping with other transition metals is extensively applied in research.^19–22^ For electrodeposited NiO_*x*_H_*y*_, Trotochaud et al. showed that just trace amounts of Fe can influence the activity of electrodeposited NiO_*x*_H_*y*_ drastically.^19^ A study by Bucci et al. tested various rock salt NiO-based catalysts synthesized through solution combustion onto Ni foam and reported that Fe contents of 40% resulted in the lowest overpotential, while Co and Mn dopants also demonstrated activity improvements.^20^ Among the factors investigated are the electronic structure^23^ and the surface reconstruction of mixed transition metal precatalysts into the active NiOOH state.^12,24–29^ In a previous publication by Mattinen et al., the varying reconstruction rates of Ni hydroxide, sulfide, oxide, and metal precatalysts toward β-NiOOH were reported.^25^ It was observed that hydroxide and sulfide exhibited similar activity and reconstruction rates. In a study by Enman et al., the number of electrons removed per Ni atom during Ni^II^/Ni^III^ oxidation was analyzed in a series of Ni-based thin films.^28^ In a recent study by Marquez et al. transition metal doping for the case of thin film NiO_*x*_H_*y*_ traces of Fe ions enhances OER electrode activity, traces of Co and Mn ions increase the capacitance, and Cu ions do not incorporate into the NiO_*x*_H_*y*_.^30^ It was proven by depth profiling, that the metal uptake is surface-confined, forming a layered structure with a saturation threshold for Fe and Co ions that maximizes electrochemical performance, where the metal uptake is depending on the solubility equilibrium of the metal ions.^30^ The results of the different studies indicated significant deviations in electron transfer between doped and pure Ni-based films with the doped materials exhibiting substantially altered electronic behavior. It can be concluded that the control of the surface structure and doping of Ni-based catalysts with Fe, Co and Mn ions represent valuable strategies for enhancing their OER activity.

A significant number of studies have focused either on one of these strategies or have been conducted on thin film model samples. However, these studies were not easily transferable to application-related powder or self-supported electrocatalysts.^31^ Moreover, this study aims to transfer the knowledge from thin film catalysts to powder catalysts with a doping approach that sustains the phase-pure and faceted precatalyst material. By this means, the combined effects of different dopants and facet control on the electrochemical properties and OER electrode activity were examined. Previously, we introduced the synthesis of NiO nanosheets with predominant (111) faceting by a microwave (MW)-assisted synthesis which resulted in reduced synthesis time and similar morphology control compared to standard solvothermal approaches.^32^ This work, on the one hand, extends the MW synthesis route to doping with other transition metals, namely, Co and Mn, enabling a phase-pure composition with a maintained NiO rock salt structure up to a doping level of 10 mol %. The effect of transition metal doping on the well-defined NiO (111) precatalyst structure, as well as the impact on the electrochemical response and OER electrode activity, is studied by X-ray absorption spectroscopy (XAS) and X-ray photoelectron spectroscopy (XPS) before and after electrochemical analysis by the rotating disc electrode (RDE) technique. While doping with more than 2% Co indicates a slightly improved OER electrode activity, the activity deteriorates for Mn-doped nanosheets. The structural peculiarities of the doping, as well as the respective activity trends for incorporation of different amounts of the transition metal ions into the faceted NiO rock salt structure, were studied, having the advantage of a well-defined host structure. The decreased charges resulting from redox changes observed in cyclic voltammetry analysis of doped samples correlate with a lesser extent of surface transformation of the rock salt precatalyst to an OER active NiOOH-terminated surface as elucidated by XPS. Consequently, the less effective reconstruction of the doped samples by electrochemical cycling could explain why the enhancement in the OER electrode activity observed with the Co-doping was just modest and the decrease in the level of the OER electrode activity observed with Mn-doping was in comparison to the pure NiO material.

## 2 Experimental Section

### 2.1 Chemicals

The following chemicals were used as received without further purification: Nickel­(II) nitrate hexahydrate [Ni­(NO_3_)_2_·6H_2_O] (Sigma-Aldrich), Cobalt­(II) nitrate hexahydrate [Co­(NO_3_)_2_·6H_2_O] (Alfa Aesar), Manganese­(II) nitrate tetrahydrate [Mn­(NO_3_)_2_·4H_2_O] (Sigma-Aldrich), urea [NH_2_CONH_2_]­(Sigma-Aldrich), benzyl alcohol [C_6_H_5_CH_2_OH] (Sigma-Aldrich), methanol pure [CH_3_OH] (Fisher Chemical), Potassium hydroxide hydrate [KOH·H_2_O] (Sigma-Aldrich, 99.995% trace metal basis), ultrapure water [H_2_O] (18.2 MΩ cm), 5 wt % Nafion dispersion (D520 1000 EW, Ion Power), Nickel oxide [NiO] (NiO_USNano_ nanopowder, 99.98%, 18 nm, cubic, US Research Nanomaterials and NiO_Roth_, Carl Roth), Lithium nickel­(III) oxide [LiNiO_2_] (Sigma-Aldrich), Cobalt­(II) oxide [CoO] (Alfa Aesar), Cobalt (II/III) oxide [Co_3_O_4_] (Alfa Aesar), Manganese (II/III) oxide [Mn_3_O_4_] (Sigma-Aldrich), and Manganese­(IV) oxide [MnO_2_] (Sigma-Aldrich).

### 2.2 Synthesis

NiO­(111) nanosheets were prepared following a procedure reported for the microwave-based synthesis approach of the same.^33^ A schematic of the synthesis approach can be found in Figure S1. Details on the MW-assisted synthesis of NiO(111) were described elsewhere.^32^ Briefly, 0.30 mol of Ni­(NO_3_)_2_·6H_2_O (1.75 g) was dissolved in 20 mL of pure methanol and stirred to obtain a light green solution. Then 0.15 mol of urea (0.18 g) was added to the solution and further stirred for 10 min. Subsequently, 0.6 mol of the benzyl alcohol (1.23 g) was added, and the resultant solution was transferred into a 35 mL microwave glass vial. The reaction was carried out at 140 °C for 30 min under stirring using the Microwave synthesizer Discover SP (CEM corporation, USA). Mn and Co doping was achieved by adding the respective molar ratio of their metal nitrates to the Ni nitrate precursor in the reaction solution. The required amounts of the Co and Mn nitrate salts are added to achieve a targeted molar ratio while keeping the total metal ion concentration at 0.3 mol. All doping levels are given as mol % in this study. The obtained hydroxides were repeatedly washed with pure methanol to remove the unreacted reagents and dried at 60 °C overnight in a vacuum oven at 250 mbar. The doped and the undoped hydroxides were subsequently calcined in a box furnace at 400 °C for 3 h at a heating and cooling rate of approximately 3 °C min^–1^. The resulting calcined nanosheets change their color from dark gray for pure NiO(111) to light brown for Co and dark brown for Mn-doped oxides.

### 2.3 Structural Characterization

The crystallinity and phase purity of the nanosheets were studied by using powder X-ray diffraction (PXRD). The PXRD patterns of the samples were measured using an Empyrean Series 2 diffractometer (PANanlytical, Netherlands) with Cu Kα radiation (λ = 0.154 nm). The PXRD patterns were recorded in θ–2θ configuration between 5 and 80° 2θ degrees.

Transmission electron microscopy (TEM) measurements were performed with a JEOL 2100FS-TEM operating at 200 kV. The materials were dispersed in ethanol under ultrasonication and drop-cast on copper grids. TEM images were taken from an 8 μm spot size with 200 ms exposure time. Energy dispersive X-ray spectroscopy mapping (EDS) was used to study the distribution of the dopant elements in one representative nanosheet after calcination, using an Oxford AZTEC EDS-system with an X-Max80 silicon drift detector.

The bulk composition was studied by inductively coupled plasma mass spectrometry (ICP-MS). For ICP-MS, 2 mg of metal oxide was suspended overnight in 2 mL of concentrated HNO_3_ (Suprapur, Carl Roth) to dissolve the powder. Then, the mixture was filtered and diluted using 2 wt % HNO_3_ to reach a final volume of 50 mL and acidification with nitric acid. A standard solution of Sc (1000 mg L^–1^ in 2% HNO_3_, Carl Roth) was added to a final internal standard concentration of 1 mg L^–1^. Co and Mn calibration solutions containing 20, 50, 100, 500, and 750 μg L^–1^ and Ni calibration solutions containing 2.0, 2.5, 3.0, 4.5, and 5.0 mg L^–1^ were prepared using a Co, Mn, and Ni ICP standard solution (1000 mg L^–1^ in 2% HNO_3_, Carl Roth). The measurement was performed using an X-Series 2 ICP-MS (Thermo Fisher Scientific GmbH). During calibration, a correlation factor of at least 0.999 was ensured, and signal intensities of the ^55^Mn, ^58^Ni, ^60^Ni, and ^59^Co isotopes were used to calculate the metal concentrations.

Nitrogen adsorption–desorption isotherms were recorded with a Tristar II adsorption setup (Micromeritics, USA). Prior to the measurements, the annealed nanosheets were degassed at 150 °C for 4 h while the as-prepared nanosheets were degassed at 90 °C overnight. Isotherms are collected between 0.005 and 0.95 *P*/*P*_0_ relative pressure, and the Brunauer–Emmett–Teller (BET) approach was followed to determine the BET surface areas.

XAS was performed at the KMC-2 beamline of the BESSY II synchrotron operated by the Helmholtz-Zentrum Berlin für Materialien and Energie GmbH.^34^ Powder samples of the as-prepared metal oxides were fixed on Kapton tape by applying the respective amounts of powder. The loaded Kapton tape was folded multiple times to an area of 1 cm^2^ to reach the desired absorption length of 1.6 for each material. The samples after electrochemical measurements (aEC) were analyzed on their respective glassy carbon (GC) discs (see the experimental details of electrochemical measurements below). X-ray absorption spectra of the Ni-K edge of the powder samples and the reference powder samples were collected in transmission mode, utilizing an ionization chamber detector. The metal foil of the particular edge (Ni, Co, and Mn) was measured as a reference in a second subsequent ionization chamber detector. The schematic of the setup for the transmission mode is shown in Figure S2a. Spectra of the dopant Co and Mn K edges of the powder samples and of the aEC-samples for all metal edges were collected in the fluorescence mode, setting the sample at 45° to a Si photodiode and the reference metal foil in the transmission configuration in front of an ionization chamber detector. The schematic of the setup for the fluorescence mode is shown in Figure S2b. The XAS energy was calibrated by setting the first inflection point of a simultaneously measured metal foil to 8333, 7709, and 6539 eV for Ni–K, Co–K, and Mn–K edges, respectively.^35^ The spectra were normalized by the subtraction of a straight line from fitting the data before the K edge and by division with a polynomial function obtained by fitting the data after the K edge for analysis of the near-edge X-ray absorption structure (XANES). The edge position of the XANES spectra was obtained by the integral method, which calculates the weighted average X-ray energy within the interval [μ_1_, μ_2_].^36–38^ The values of edge positions were utilized to analyze qualitative trends in the oxidation state with doping and electrochemical treatment. The optimized intervals [μ_1_, μ_2_] for the Ni–K, Co–K, and Mn–K edges are [0.15, 1], [0.2, 1], and [0.25,1], respectively. The Fourier transformation (FT) of the EXAFS was calculated between 35 and 360 eV (3.03 to 9.72 Å^–1^) for the Co-K edge, Mn-K edge, and Ni–K edge after the K edges. A cosine function was used to repress the sidelobes of the FT. Data processing was done using the in-house software BESSY 4.0.

The surface composition and chemical state of the materials were analyzed by XPS measurements with an ESCALAB 250 Xi device (Thermo Fisher, UK) with a Mg X-ray source (*hv* = 1253.6 eV). High-resolution spectra of Ni 2p, Co 2p, Mn 2p, O 1s, and C 1s were recorded at a pass energy of 10 eV with a step size of 20 meV. The adventitious carbon C 1s signal at 284.8 eV is used as a charging reference. The spectra were analyzed using the Avantage software version 4.97, and peak deconvolution of the O 1s and C 1s spectra was performed using a Shirley background.

### 2.4 Electrochemical Characterization

The electrochemical tests in a three-electrode setup were performed by using a PTFE cell filled with 100 mL of 0.1 M KOH electrolyte. Exchangeable GC RDEs by Pine Research (5 mm disc diameter, AFE6R1PTPK) were mounted to a Pine Research MSR Rotator connected to an Autolab PGSTAT128N (Metrohm) operated with Nova 2.1 software. Commercial GC discs from HTW (SIGRADUR G, 5(±0.05) mm, polished and lapped) were used as substrates. A graphite rod and a mercury/mercuric oxide electrode (Hg/HgO, RE-6A, ALS Co.) were used as counter and reference electrodes, respectively. The latter one was calibrated against a reversible hydrogen electrode (RHE) consisting of a platinum disc electrode (AFE7R8PTPT, Pine Research) in a H_2_-saturated 0.1 M KOH electrolyte. All experiments were conducted under ambient conditions.

Prior to the coating, the RDE was polished with 1 and 0.05 μm alumina powder (MicroPolish, Buehler) slurry with subsequent ultrapure water washing for 3 min each. After polishing, the alumina residues were removed by sonication of the RDE in isopropanol and DI water for 2 min, respectively. The polished RDE was dried under N_2_ stream and mounted to a rotator for drop casting of the catalyst suspension. The suspension consisted of 4 mg of metal oxide, 1587 mg of DI water (1590 μL), 312 mg of isopropanol (400 μL), and 8.6 mg of Nafion D-520 Dispersion (9.04 μL, 5 wt %, Sigma-Aldrich). The suspension was horn-sonicated (Digital Sonifier 250, Branson) for 10 min at 10% intensity (25 W) with 10 s on and 5 s off pulse program in an ice bath cooling. After homogenization, 9.82 μL of the suspension was dropped onto the RDE while rotating at 100 rpm leading to approximately 100 μg cm^–2^ of catalyst loading. The RDE was dried in air by rotating at 700 rpm.

Before the electrochemical characterization, the electrolyte was purged with N_2_ for 30 min, while the electrode was rested in the electrolyte at open circuit potential (OCP). The double-layer capacitance of the electrode was tested in a N_2_-purged electrolyte. Therefore, linear sweeps with several scan rates between 5 and 500 mV s^–1^ were performed in a potential window optimized for NiO(111) between 0.9 and 1.1 (positive going) and 1.1–0.9 (negative going) V vs RHE. For estimation of the capacitance, the current was taken from 0.93 V vs RHE of the negative going scan for the different scan rates ν. The capacitance was taken as slope from an allometric fit of *i* vs ν with the formula *i* = *C*_DL_ν^α^ with the exponent α describing the discrepancy of the plot to the linear model of an ideal capacitor (α = 1). For further electrochemical tests, the electrolyte was purged with O_2_ for 15 min. The electrolyte resistance *R*_u_ was measured by electrochemical impedance spectroscopy (EIS). This was used to correct the Ohmic drop of the applied potential by 100% in the postprocessing by subtracting it with the product of the current *i* and the electrolyte resistance *R*_u_. The activity was determined by cyclic voltammetry at 2500 rpm with a scan rate of 10 mV s^–1^ before and after electrochemical cycling. For conditioning, the electrodes were electrochemically cycled with 350 CV at 100 mV s^–1^. Details on the measurement procedure can be found in the Supporting Information in Table S1. The potential is referred vs RHE. All materials were tested three times with freshly prepared electrodes.

## 3 Results and Discussion

### 3.1 Physical Characterization

The pure NiO nanosheets with a predominant (111) surface faceting (NiO(111)) were prepared by a previously described MW-assisted synthesis with a subsequent calcination step, where the effect of the calcination temperature on the catalyst’s structure and OER electrode activity was studied.^32^ The MW synthesis route is a rapid and adaptable method that can be employed as an alternative to an autoclave synthesis approach.^33^ Both methods utilize urea and benzyl alcohol as a base and as a structure-directing agent, respectively, in order to obtain predominantly NiO(111) nanosheets. Optimized synthesis parameters of the prior study with a calcination temperature of 400 °C and a synthesis time of 30 min have been adopted here as optimum between the overpotential and the synthesis time.^32^ Doping levels of Co and Mn were primarily varied between 2 and 10 mol % and will be denoted as NiO(111) + *x*% M (M = Co, Mn), with *x* being the targeted molar ratio of dopant (e.g., NiO(111) + 5% Co for the material with 5% Co and 95% Ni molar mixing ratios used during the synthesis). The apparent metal contents of the powder samples were measured with ICP-MS after dissolution in nitric acid. The apparent doping levels were very close to the weighed portions, except for higher Co doping levels (>20% Co doping) and are compiled in Table S2.

The crystal structure and phase purity after doping were investigated using PXRD, which revealed the presence of NiO rock salt and no other crystalline phases (Figure 1). The reflexes at 2θ of 37.1° were assigned to the (111) facet, the reflexes at 43.2° 2θ were assigned to the (002) facet, and the following reflexes at 63.6, 75.3, and 79.3° 2θ were assigned to the (220), (311), and (222) crystal planes of the face-centered cubic type NiO with a space group of Fm3m (PDF 98–018–4918). For metal doping, a shift of distinct reflexes was reported due to various atomic radii of the metals that result in a change of the lattice parameters.^20^ A closer look at the (002) reflex reveals the influence of the second metal incorporation into the lattice structure. Slight shifts to lower 2θ angles were observed for the Co-doped materials in Figure 1b), which are expected by larger lattice parameters of CoO with 4.261 Å compared to NiO with 4.195 Å.^6^ However, for the Mn-doped materials, a shift to higher 2θ angles in Figure 1d was observed. Because of previously reported lattice parameters of 4.46 Å for MnO, a shift to lower angles would be expected.^39^ Both observed shifts indicate a minor structural effect for the low doping levels. However, the magnitude of the observed shifts as well as the peak position of the 5% samples, which are opposite to the trend of the other doping levels, is in the range of experimental error. One possible explanation is that the ionic radii of the metal ions Ni^II+^ and Mn^II+^ are similar, with values between 0.65 and 0.69 Å, respectively.^40^ The phase pure doping of Co into the NiO rock salt in an autoclave synthesis followed by calcination was previously described to result in shifts of the (222) facets to lower 2θ angles for doping levels higher than 30% of Co.^6^ Bucci et al. reported a combustion synthesis of 10% doped NiO together with various transition metals which resulted in different mixed metal oxides with characteristic NiO rock salt reflexes, each with the (002) reflex at 43° shifted to lower 2θ angles.^20^ For the Co-doping, higher doping levels until 60% Co were synthesized, but PXRD revealed phase impurities of presumably Co_3_O_4_ besides the rock salt phase and strong shifts toward higher 2θ angles were observed. The PXRD results of the higher Co-doped oxides is depicted in Figure S3). The results align with observations of Co_3_O_4_/CoO that were described for oxidation of pure Co at different conditions.^41^

**Figure 1:**
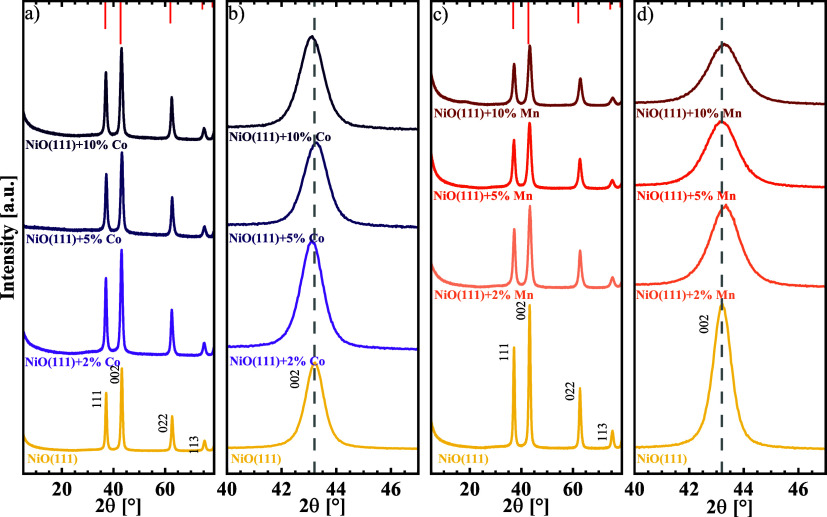
Powder X-ray diffraction patterns of (a, b) Co and (c, d) Mn doped NiO nanosheet with (b) and (d) showing a zoom into the (002) reflex. Both dopants are compared to NiO(111). The respective 2θ angles of space group *Fm*3*m* (PDF 98–018–4918) are displayed on the upper axis.

The Scherrer Equation was used to estimate the crystallite sizes. Both Co- and Mn-doped nanosheets exhibit a smaller crystallite size compared to the respective undoped nanosheets (Table 1). Note that the Mn-doped samples have a relatively smaller crystallite size (6–7 nm) than the Co-doped counterparts (9–10 nm).

**Table 1:** Crystallite Sizes from the Scherrer Equation Calculated for the (002) PXRD Signal and Surface Area of NiO Nanosheets with Different Doping Levels From BET Analysis of Nitrogen Physisorption Experiments

material	crystallite size (002) [nm]	BET surface area [m^2^ g^–1^]
NiO_USnano_	33.3	28
NiO(111)	11.0	71
NiO(111) + 2% Co	9.7	85
NiO(111) + 5% Co	8.9	86
NiO(111) + 10% Co	9.1	62
NiO(111) + 20% Co	8.0	52
NiO(111) + 2% Mn	6.8	107
NiO(111) + 5% Mn	6.4	107
NiO(111) + 10% Mn	6.1	115

TEM images in Figure 2 were acquired to study the nanosheet structures of the different catalyst materials. All the TEM images demonstrate the preservation of the nanosheet morphology with hexagonally formed pores, as previously described for the original NiO(111) material.^32,33^ The observed NiO(111) + 5% Co nanosheet has comparable pore sizes to the pure NiO(111) sample in the range of tenths of nanometers from the TEM images. The overall shape of the nanosheets appears to be slightly influenced by doping. The observed NiO(111) + 5% Mn nanosheets appear folded or stacked with pores in the low nanometer range, which are smaller compared to the other nanosheets. The energy dispersive spectroscopy (EDS) mapping of the doped nanosheets is shown in Figure S4 and indicates a homogeneous distribution of the dopants (Co and Mn) in the NiO studied nanosheets. No agglomerations of dopant elements were found from EDS elemental mapping, which would have indicated the formation of separate phases, as indicated in other studies.^42^

**Figure 2:**
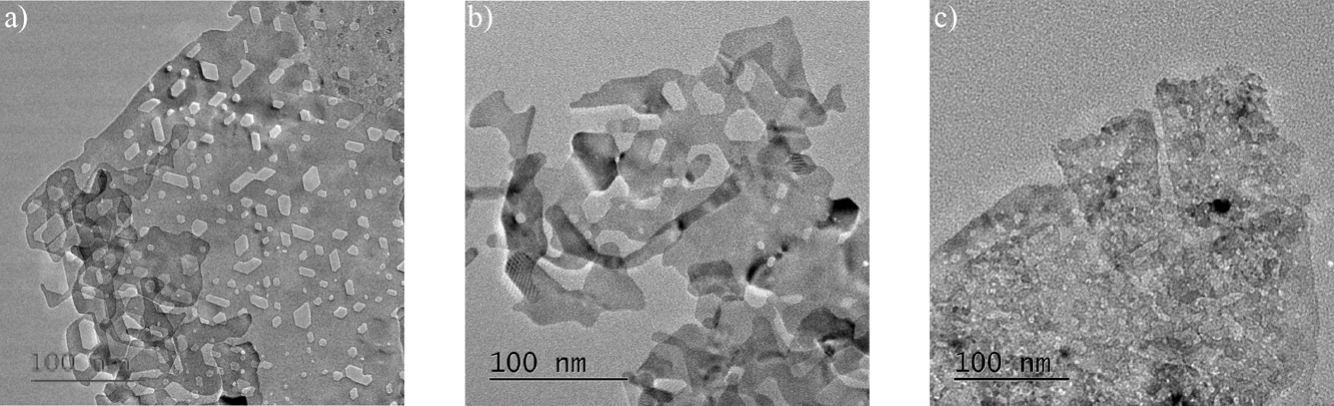
Transmission electron microscopy images of undoped NiO(111) in (a) and NiO(111) + 5% Co in (b) as well as NiO(111) + 5% Mn in (c).

The surface areas of the undoped and doped NiO were investigated using nitrogen adsorption–desorption experiments. The adsorption–desorption isotherms are plotted in Figure S5 and were fitted with the BET model to calculate the surface area of the NiO(111)-based materials and of a commercial NiO_USnano_ reference material used for electrochemical evaluation (Table 1). Differences in the specific surface areas between the doped and undoped materials were observed. Compared to pure NiO(111), the Mn doping increased the surface area from 71 m^2^ g^–1^ up to 115 m^2^ g^–1^ for the NiO(111) + 10% Mn sample. Correlating that to the small crystallite size and TEM images, the increased BET surface area might be a result of the increased number of pores in the sheets. Co doping up to a level of 5% led to an increase in the BET area; however, higher doping levels >10% seem to lower the surface area to 62 m^2^ g^–1^ for the NiO(111) + 10% Co. To confirm this trend, a Co doping level of 20% was included in the analysis, showing a further decrease in the BET surface area. This observation correlates with the formation of Co_3_O_4_ phases at doping levels higher than 20% of Co.

The physical characterization above suggests that the analyzed doping levels lead to phase-pure rock salt NiO nanosheets for both the Mn and the Co doping. The Mn doping moderately affected the pores of the NiO(111) + 5% Mn, which may lead to an increased apparent BET surface area. The influence of the low Co doping levels up to 5% on the structure was not evident as TEM images and BET surface area were close to those of the pure NiO(111) sample. In contrast, Co doping levels of 10 and 20% resulted in lower BET surface areas and decreased crystallite sizes. In general, high surface areas are expected to improve the electrocatalytic performance of metal oxide-based OER electrocatalysts^43^; however, the BET surface area might not reveal the electrochemically active surface area (ECSA), which is more relevant, but difficult to obtain for metal oxides. The electrochemical behavior and the OER electrocatalysis will be the focus of the following section.

### 3.2 Electrochemical Characterization

The electrochemical characterization, as well as determination of the OER electrode activity, was performed in a 0.1 M KOH electrolyte by the RDE technique. Details of the electrochemical measurement protocol can be found in Table S1 and are derived from recommendations by Anantharaj and Noda.^44^ After initial OER electrode activity measurements, the materials were cycled 350 times in a potential window from 1.0 to 1.7 V vs RHE at 100 mV s^–1^ sweep rate to reach a stable cyclic voltammogram (CV) with subsequent OER activity measurements of the activated sample. A cycle number of 350 scans was chosen to reach a stable current response of the Ni^II^/Ni^III^ oxidation peak of the pure NiO(111) material. During cycling, the surface structure of the NiO(111) catalyst will change forming OH/OOH functionalities as the active phase for the OER. Thus, CVs of the activated materials with 10 mV s^1^ under OER conditions were used to compare the OER electrode activity. It has to be stated that high-purity KOH was used for the preparation of the electrolyte, but no further purification was done. The Fe content determined by ICP-MS (Table S3) in the electrolyte of around 1.5 μg L^–1^ (1.5 ppb) by ICP-MS is considered negligible in comparison to studies using intentional doping with 100 ppb or higher levels. The developments of consecutive CVs in Figure 3a were analyzed to gain insights into structural differences between the pure NiO(111) and materials, with an intermediate doping level of 5% Co and Mn as representative samples. Results of the 10% samples are plotted in Figure S6 and support the results of the 5% samples. Recently, the different incorporation modes of, for example, Ni, Co, and Fe ions into Ni- and Co-based thin films were demonstrated by emphasizing the characteristics of the CV measurements.^26^ In the case of pure NiO(111), the electrochemical cycling resulted in an increasing Ni^II^/Ni^III^ redox peak from 1.4 to 1.6 V vs RHE with a charge of 127 C g^–1^ of the last 350th CV. This behavior was previously described by the growth of the surface (oxy)­hydroxide layer by electrochemical conditioning due to the oxidation from Ni^II^(OH)_2_ to Ni^III^OOH.^45,46^ The peak position of the NiO(111) sample remained unchanged during the cycling. For comparison to the broader literature, the cycling was also performed with 50 mV s^–1^ and the NiO(111) sample in Figure S7. A higher activation was observed at 50 mV s^–1^.

**Figure 3:**
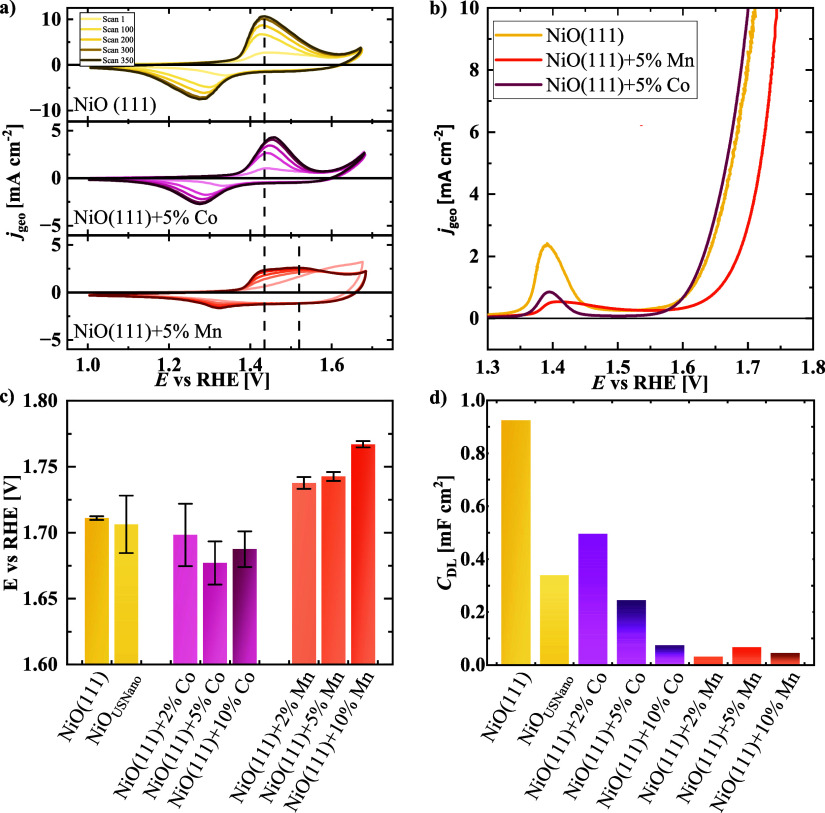
(a) Representative cyclic voltammograms of the pure NiO(111) and the 5% Mn- and Co-doped samples, with a dotted line at 1.43 and 1.52 V vs RHE as a guide to the eye, (b) positive going linear sweep voltammetry scans of NiO nanosheets without dopants and 5% doped samples, (c) overpotentials at 10 mA cm^–2^ for different doping levels as metric for the OER electrode activity, and (d) double layer capacitance from linear sweep voltammograms at different scan rates. Supplementary electrochemical data can be found in the SI.

Doping of NiO with further transition metals results in lower current density of the Ni^II^/Ni^III^ redox peak and a change of the position in CV measurements, which was previously observed for various Ni-based mixed metal electrocatalysts.^28,46,47^ The NiO(111) + 5% Co sample behaves comparable to the pure NiO(111) with regard to the redox peak position and intensity with increasing cycle number. The Ni^II^/Ni^III^ redox peak is shifted to higher potentials with increasing cycle numbers. The absence of the Co^II^/Co^III^ redox peaks indicates an integration of Co ions into the NiO lattice and the formation of a mixed surface hydroxide layer.^26^ However, the Co-doped sample has a nonproportionally smaller Ni^II^/Ni^III^ oxidation peak charge of 41.0 C g^–1^, which is about 1/3 in comparison to the pure NiO(111) sample. The lower Ni^II^/Ni^III^ oxidation peak charge of the Co-doped samples can be explained by a suppression of the formation of NiOOH species.^46^ However, the reason for the suppression of NiOOH formation could not be resolved.

In the case of the NiO(111) + 5% Mn sample, the charge of the Ni^II^/Ni^III^ oxidation peak of the 350th scan further decreased to 31.3 C g^–1^, which is about 1/4 compared to that of pure NiO(111) for the same catalyst loading. CVs of the NiO(111) + 10% Co and Mn samples in Figure S6 have similar trends in the Ni^II^/Ni^III^ redox peak charge as the 5% samples with even lower oxidation charges. The strong deviation between dopant content and Ni^II^/Ni^III^ redox peak charge could result from a lower accessibility of surface Ni^II^(OH)_2_, for example, by a low conductive phase, which led to challenges in extracting the in-plane conductivity of Mn-based oxy-hydroxides in a previous study.^48^ Furthermore, a shift in the position of the Ni^II^/Ni^III^ oxidation peak position of the first CV from 1.43 V vs RHE of the pure NiO(111) toward higher potentials of 1.6 V vs RHE of the NiO(111) + 5% Mn sample is observed. This shift in the first CV could be explained by an electronic influence of the Mn dopant on the NiO(111) host material, which was observed for the incorporation of Fe into electrodeposited NiO_*x*_H_*y*_ films.^19,26^ Furthermore, this peak shift is only observed for bulk incorporation (e.g., by CV) and not for surface-restricted incorporation by chronopotentiometric measurements.^27^ The effect of the Mn doping on the initial oxidation peak position is evidence of Mn being incorporated initially into the NiO lattice. However, after the first 100 cycles, a splitting of the oxidation peak is observed, resulting in two overlapping peaks at about 1.43 and 1.52 V vs RHE. Distinct peaks in this region were found previously and were attributed to tetrahedral and octahedral metal species in spinel-type transition metal oxides.^49,50^ More likely for these samples, the different signals arise from redox changes of Mn species on the surface, in addition to Ni redox processes. Mn oxidation in this potential regime was observed to lead to dissolution as MnO_4_^–^.^37,51^ To detect Mn corrosion, generation-collection experiments of the NiO(111) + 10% Mn sample were performed with the RRDE setup in an N_2_-saturated electrolyte. Figure S8 shows oxygen detection at the ring electrode by applying a potential of 0.4 V vs RHE. The corrosion of Mn was probed with a ring potential of 1.2 V vs RHE, which was previously described for a LiMn_2_O_4_ catalyst.^37^ However, the experiment in Figure S9 suggests a slight reduction of MnO_4_^–^ ions at the disc potentials of 1.7 V vs RHE and beyond. Complementary ICP-MS experiments in Table S4 have detected a concentration of 0.902 μg L^–1^ of Mn in the electrolyte after the generation-collection experiments of the 10% Mn sample from Figures S8 and S9. This implies a leaching of about 7% of Mn out of the catalyst layer compared to about 0.6% of the Ni. Therefore, it can be concluded that the dissolution of Mn is too low to be detected by RRDE generation-collection experiments, particularly given the low overall Mn loading, but ICP-MS results strongly support the Mn leaching, especially from oxidation processes.

In other studies, the surface redox reaction was used to estimate the ECSA of the catalyst.^43^ Herein, the studied multimetal systems with their multiple redox changes limit the applicability of this technique. Therefore, we did not evaluate the surface area from the redox charge. Apart from that, it was reported that the specific charge of the Ni^II^/Ni^III^ redox peak of Co-doped NiO nanoparticles in the rock salt structure decreased continuously with increasing Co content: from 231 C g^–1^ down to 68 C g^–1^ at 70% Co doping, which was about 30% of the value of the undoped NiO.^6^ These observations of a linear correlation between dopant content and the Ni^II^/Ni^III^ oxidation peak charge are contradictory to the present study. This could be explained by the two different synthesis methods, which are the microwave synthesis compared to a solvothermal colloidal synthesis and the different particle morphologies, for the case of faceted nanosheets and nanoparticles. Additionally, differences in the electrochemically active surface area due to conductivity or the specific surface area could influence the oxidation peak charge tremendously.^48^ Thus, the discrepancy between the studies indicates an effect of the different catalyst morphologies on the properties of doped Ni oxides. Consequently, a detailed analysis of redox changes of different dopants and doping levels dependent on different catalyst morphologies could help to understand the nature of doping.

As a metric of the OER electrode activity,^52^ the overpotential at a geometric current density *j*_geo_ = 10 mA cm^–2^ was extracted from the positive going scan after activation (Figure 3b) and overpotential values are plotted in Figure 3c and documented in Table S6. Data from the repetition experiments are shown in Figures S10–S12. Doping levels of 2, 5, and 10% of Co and Mn were compared regarding their OER electrode activities. For benchmarking, a commercially available NiO_USnano_ nanopowder was used. The lowest overpotential was measured for the NiO(111) + 5% Co with an overpotential of 447 ± 16 mV, obtained from three independent experiments. The sample NiO(111) + 5% Co reduces the average overpotential by 34 mV compared to 481 ± 1 mV for pure NiO(111). Generally, a decrease in overpotential is a consistent finding in the majority of studies examining Co-doping in nickel-based OER electrocatalysts.^20,30^ In comparable experiments of Co–Ni based OER catalysts, overpotentials of about 360 to 470 mV were found.^3,53^ The sample with NiO(111) + 10% Mn even increases the overpotential to 537 ± 3 mV. There are many studies in which Mn worsens the OER electrode activity after addition to Ni-based electrocatalysts.^3,54,55^ In the work of Dionigi et al., the negative effect of Mn increasing the OER overpotential at 10 mA cm^–2^ from 570 mV for pure Ni­(OH)_2_ to 620 mV for NiMn LDH was explained with the aid of density functional theory calculations of single-phase γ-NiMn LDH showing an increase of the reaction free energy of the OER due to Mn compared to γ-Ni LDH.^3^ On the other hand, examples of positive influence of Mn for the OER of Ni-based systems were also reported.^20,56^ An explanation for this contradiction might be the variety of synthetic approaches and host structures, for example, LDH from autoclave precipitation, rock salt oxides from a solution combustion synthesis, and oxides from a colloidal synthesis^3,20,55^ that could influence the chemical state of Mn and phase purity of the sample. The overpotentials of samples with Co doping levels up to 60 mol % are shown in Figure S14. In general, the higher doping levels neither show a clear trend nor higher overpotentials than, for example, the activated NiO(111) + 5% Co sample. The overpotential slightly increases with Co doping levels between 10 and 60%, where electrochemical cycling resulted in a decreased OER electrode activity compared to the initial state. The higher Co-doping levels have shown impurities of Co_3_O_4_ in the XRD, which can contribute to the deactivation of the catalyst by cycling due to a hindered reconstruction of the spinel material into the hydroxide material.^46^ The repeatability of the experiments, which was measured with the standard deviation between the independent experiments, reveals differences in the repeatability between the Co-doped samples and the commercial NiO material, which had large deviations in the overpotential at *j*_geo_ = 10 mA cm^–2^ in the range of 14 mV up to 24 mV (Figure 3c). We attribute this to differences in the dispersibility of the catalyst materials.^57^ However, no macroscopic differences were visible regarding the ink stability and the homogeneity of catalyst films. On the other hand, the pure NiO(111) and the Mn-doped materials had acceptable standard deviations in the range of 1–5 mV. Furthermore, the influence of possible Fe contaminations on the structural and electronic properties of reconstructed NiOOH films needs to be considered. It was assumed that the influence of Fe on each sample was comparable. Additionally, activity measurements at varying scan rates are represented in Figure S13 after cycling at different scan rates and in 1 M KOH electrolyte to compare the results to the wider literature. The activity was the highest after cycling with 50 instead of 100 mV s^–1^.

The double layer capacitance (*C*_DL_) as surface-originated metrics of the materials was measured by linear sweep voltammetry at different scan rates, and the current window was optimized for pure NiO(111).^58^ The respective voltammograms are shown in Figures S15–S18. The pure NiO(111) sample had a *C*_DL_ of 0.9 mF cm^–2^, being the highest value in the comparison. This value is substantially higher compared to 0.07 mF cm^–2^ for a NiFe-based LDH measured by the RDE experiment with a comparable catalyst loading and setup.^47^ Priamushko et al. determined the capacitance of their NiO films with 120 μg cm^–2^ catalyst loading on a GC substrate to be 82 μF, hence 0.42 mF cm^–2^ in 1 M KOH.^49^

The CV traces of the doped NiO were less box-like in the considered potential window, which was too narrow for full charge/discharge, for some cases reducing the determined capacitance and might include faradaic metal redox response, which increases the determined capacitance.^58,59^ Thus, we focus on trends in the measurement series. The Co doping decreases the *C*_DL_ with increasing doping level. The lowest value was obtained for the NiO(111) + 10% Co sample with 0.075 mF cm^–2^. The Mn samples exhibit a further reduced *C*_DL_ values; however, they do not reveal a trend of the doping onto the *C*_DL_, because the NiO(111) + 2% Mn and +10% Mn samples have similar *C*_DL_ values of 0.031 and 0.045 mF cm^–2^. Thus, all doped NiO(111) showed a clear reduction of *C*_DL_ by at least 90%. Furthermore, the drop in *C*_DL_ agrees with the drastic lowering of the Ni^II^/Ni^III^ redox peak intensity but is contradictory to the BET surface areas, which showed an increased surface area of doped samples, especially for Mn-doped materials. This suggests that most of the increased physical surface area of the doped samples observed from physisorption experiments might not be electrochemically accessible for nonfaradaic (*C*_DL_) or faradaic charge transfer (Ni^II^/Ni^III^ redox peak). To categorize the *C*_DL_ data, the conductivity of the Ni-based catalyst of transition metal-based oxide and hydroxide precatalysts should be mentioned. The conductivity of the catalysts strongly depends on the oxidation state of the catalyst, which is influenced by the potential at which the experiment is performed.^60^ The conductivity of the materials will also have an effect on the electrochemically accessible surface area and might underestimate the surface area. A previous study on NiO*_x_-*based transition metal oxides suggests an improved conductivity of the material after mixing it in binary (NiCoO_*x*_) and ternary materials (NiCoFeO_*x*_) with Co.^61^ Another study, which is based on electrodeposited hydroxide films, has suggested that MnO_*x*_H_*y*_ films are poor electrical conductors, which might explain the observations of the low *C*_DL_ and Ni^II^/Ni^III^ redox peak charge in the present study.^60^ Additionally, the α-value was received, which resembles the deviation of the regression from a linear fit and thus from the ideal behavior of a capacitor, and is shown in Table S5 for the respective materials.^58^ The pure NiO samples have a similar high α-value close to 0.9, which is expected because the chosen potential window for the experiment was optimized for this material. However, the doped materials exhibit lower α-values with an increasing doping level. This implies that the addition of further transition metals decreases the ideal capacitive behavior of the metal oxides in the respective potential window.

Different electrocatalytic metrics for the electrode activity naming η at 10 mA cm^–2^, the BET current density *j*_BET_ normalized to the specific surface area from BET at 1.65 V vs RHE and the current density normalized to *C*_DL_ *j*_CDL_ at 1.65 V vs RHE were compiled in Table S6 and Figure S19 to relate the material properties to their activity. The commercial NiO_USNano_ with 142 nA cm_BET_^–2^ is the highest BET-related current density, combining a moderate overpotential and a low BET surface area. On the other hand, the 10% Mn-doped material combines high surface areas and increased overpotentials, resulting in the lowest BET current densities with 8.71 nA cm_BET_^–2^. The material with the lowest η NiO(111) + 5% Co exhibits moderate BET- and *C*_DL_ *-*normalized current density. The reported metrics have only limited liability if intrinsic properties, such as the specific surface areas from BET and *C*_DL_ are contradictory. To further elucidate the effect of the dopants on the chemical state and match it to the electrochemical response and OER electrode activity, XAS and XPS were performed and will be discussed in the following.

### 3.3 Ex Situ Spectroscopic Characterization

We performed XAS investigations before (bf) and after electrochemistry (aEC) to further understand the influence of Co and Mn doping on the bulk oxidation state and structure to correlate it with the OER electrode activity of the samples. Note that bf-samples of XAS studies were obtained from pristine powder samples, while aEC-samples of XPS and XAS studies were obtained from processed films on GC disc electrodes after the electrochemical protocol was applied (Table S1). Trends in the oxidation state were qualitatively obtained from the edge position determined as the centroid of the normalized XANES spectra, which are shown in Figure S20 and whose edge energies are documented in Table S7.^36,37^ The structures of the materials were determined by comparing the EXAFS structure of the samples with suitable references. Insights from the Ni–K, Co–K, and Mn–K edges are compared below; a comprehensive picture of the influence of doping and their changes due to the OER are presented in Figure 4.

**Figure 4:**
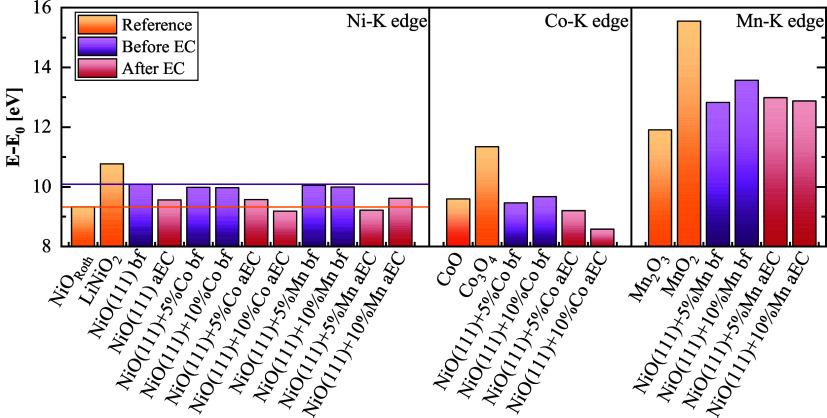
Normalized edge positions *E* – *E*_0_ from XANES of the Ni–K edge, Co–K edge, and Mn–K edge for different dopants and doping levels before and after EC (aEC). Reference lines are drawn to the *E* – *E*_0_ of Ni^II^O_Roth_ in yellow and that of NiO(111) in violet.

In the following, the position of the edge energy E relative to the corresponding metal edge energy *E*_0_ will be discussed. First, the impact of doping on the as-prepared powders (bf samples) was investigated. The initial Ni-K edge position of undoped NiO(111) fell between the edge positions of the references NiO_Roth_ and LiNiO_2_ with respective nominal oxidation states of Ni^II^ and Ni^III^. This suggests that the initial oxidation state of the Ni was between 2+ and 3+. Calculating the average of the Ni^II^ and Ni^III^ references gives an edge position close to that of NiO(111), from which an oxidation state of 2.5+ is estimated and highlighted as a guide to the eye in the bar chart (Figure 4). No change in the edge position and thus Ni oxidation state was found for samples doped with either Co or Mn at levels of 5 and 10%. At the Co–K edge of the Co-doped NiO(111), there was no significant variation, and the edge position was shared with the Co^II^O reference. At the Mn–K edge of the Mn-doped NiO(111), both samples had an edge position between the Mn^III^ and Mn^IV^ references with that of 10% Mn doping higher than 5% doping. Interestingly, the edge position at the Mn–K edge depended on doping, while the edge position at the corresponding Ni–K edge did not. Because there is no indication in the XANES or the EXAFS that the coordination environment of the metal site changes, we rationalize this by a change in the oxygen stoichiometry. Qualitatively, the FT of EXAFS (Figure 5) supports higher oxygen coordination with 10% Mn doping relative to 5% Mn doping that is expected for the higher metal oxidation state due to charge compensation.

**Figure 5:**
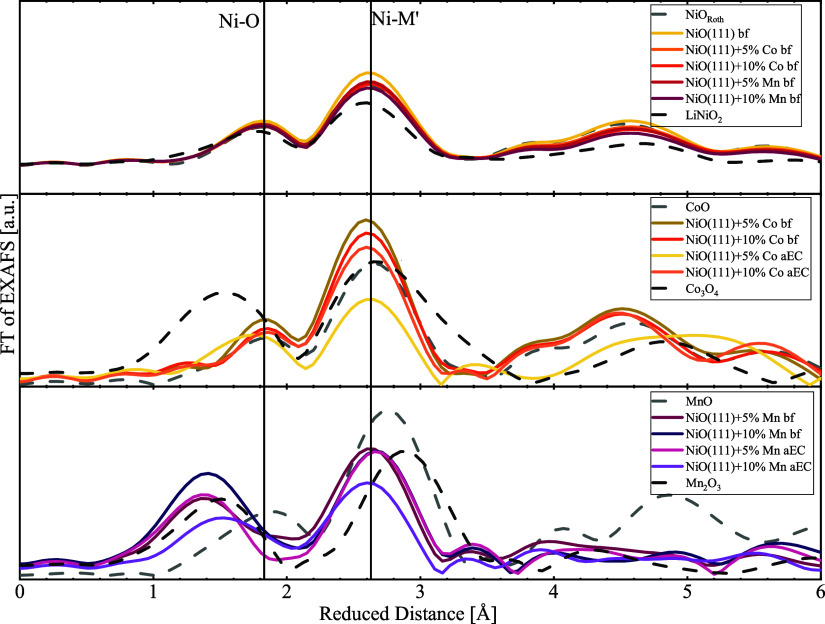
Fourier transformation of the Ni-, Co-, and Mn–K edge EXAFS for the prepared samples and reference materials. In each graph, the reduced distance between Ni-M’ as well as the Ni–O from the Ni–K edge is marked.

Next, it was studied how the electrochemical treatment affects the observed edge positions relative to the as-prepared samples because the electrochemically treated samples are a better approximation of the active state during electrocatalysis than the as-prepared samples.^62^ After the OER, both the undoped and doped Ni(111) samples show a lower edge position at the Ni–K edge, indicating a reduction relative to the as-prepared powders. The edge position of the samples is similar to within 0.3 eV to that of the Ni^II^ reference indicated as the reference line in the bar chart. At the Co–K edge of the Co-doped samples, there was no significant change relative to the as-prepared powder for 5% Co doping, while the 10% Co doping sample showed an edge position lower than that of the respective as-prepared powder and lower than the Co^II^ reference. At the Mn–K edge of the Mn-doped samples, the trends of the edge position are comparable to that at the Co–K edge, namely, no significant change for 5% doping and a reduction of the edge position for 10% doping, where, however, the edge position remained above that of the Mn^III^ reference. We hypothesize that the oxygen stoichiometry is equalized among the differently doped samples due to the formation of the active OER state. Furthermore, the Mn^IV^ species in the Mn-doped material may introduce strain and electronic distortion into the coordination environment and contribute to the instability of the Mn materials which was observed above.^30^ This would explain Mn^III^ reducing Ni^III^ to Ni^II^, leading to the oxidation of Mn^III^ to Mn^IV^ and the reduction to Ni^II^ observed for the aEC samples. In summary, the edge position was lower than or equal to the as-prepared powders. While a lower edge position is commonly interpreted as a reduction in oxidation state, this is only correct without significant variation of the coordination environment.^62^ Thus, we turned to the analysis of the EXAFS to qualitatively evaluate whether the low edge positions are rooted in an unusual coordination environment.

The coordination environment around Ni, Co, and Mn was studied by EXAFS analysis (Figure 5). The coarse position and relative peak heights align well with those of the respective MO references for Ni and Co, confirming that the bulk structure is consistent with rock salt MO, as supported by PXRD analysis. We note that rock salt NiO and layered LiNiO_2_ cannot be easily distinguished at the Ni–K edge within the experimental noise and sampled k-space information (<12 Å^–1^). Thus, the hypothesized surface hydroxide cannot be resolved in our experiments, and we turn to XPS analysis below to elucidate it. At the Mn–K edge of the Mn-doped samples, the Mn-M peak position of Mn-doped NiO(111) aligns better with the Ni-M position of the same sample at the Ni–K edge as compared to the Mn–Mn position of MnO, while the Mn–O peak was near that of the Mn^III^ and Mn^IV^ references. There is no evidence of separate phase formation of Mn_3_O_4_ and MnO_2_ in Figure S21. At the Co–K edge of the Co-doped samples, the 5% doped sample showed Co–O and Co–Co peaks aligned with CoO, suggesting phase separation of Co. In contrast, the 10% doped sample had those peaks aligned with the Ni–O and Ni-M peaks at the Ni–K edge of the same sample. First, these observations support that Co with a 10% doping level and Mn were doped in the Ni matrix. Second, the unusually low edge position of the 10% Co-doped sample below the +2 reference might be rooted in the untypical bonding environment compared to the cubic Co^II^O­(II), although more experiments, which are beyond the scope of this report, would be needed to rigorously elucidate this point. Third, the edge shifts at the Mn–K edge may also not be quantitatively converted into a change in the Mn oxidation state as Mn also experiences the bonding environment of the Ni matrix, yet qualitatively, the magnitude of the edge position is higher than that of Co, indicating oxidation possible to above Mn^III^. Metal sites forced to untypical bonding environment can lead to a catalytic enhancement as discussed for octahedral Fe sites with shortened Fe–O distances in an Ni_1–*x*_Fe_*x*_OOH,^63^ which may explain the slight decrease of the overpotential in Figure 3 with Co doping, while for Mn doping Mn^4+^ was consistent with the high edge position, which is known to increase OER overpotentials.^37,64^ We note that high valent metal cations may be lost to the electrolyte, particularly for Mn as discussed and tested in the RDE section above.^37^ Finally, neither Ni^II^ nor Co^II^ is known to be a high-performance active site,^65–67^ but they are frequently oxidized on the surface, which was probed by XPS.

In addition to the redox changes of the metal centers probed by bulk-sensitive XAS measurements, surface-sensitive XPS measurements were performed. The XPS spectra were recorded to gain insights into the surface changes by electrochemical treatments as before electrochemistry (bf) samples and as after electrochemistry (aEC) samples by removing the coated GC disc substrate electrode at a stop potential of 1.7 V vs RHE out of the electrochemical cell. The survey spectra in Figures S24–26 of the pristine bf-samples indicate the presence of the expected elements. Hence, the NiO(111) bf-sample shows O, Ni, C, F, S, and X-ray satellite peaks around the most dominant signals like Ni 2p and F 1s from the non-monochromatic Mg Kα source. Additionally, for Co- and Mn-doped samples, the corresponding dopant elements were also found in the survey spectra. Due to complex multiplet splitting of the transition metal 2p spectra, peak deconvolution was only performed for the high-resolution O 1s and C 1s spectra, while Ni 2p, Co 2p, and Mn 2p are qualitatively analyzed.

Figure 6a shows the Ni 2p_3/2_ spectra of NiO(111) and the corresponding 5% Co and Mn doped bf-samples. The Ni 2p_3/2_ spectra are similar for all three samples, indicating a very similar surface composition and chemical state of Ni atoms in accordance with XANES analysis of the Ni-K edge. Thus, the low doping level has a rather low impact on the surface-sensitive XPS signal. The Ni 2p spectra show the characteristic multiplet splitting of NiO with a dominant signal at a binding energy of 854.2 eV and a less intense signal at 855.9 eV.^68^ The first signal at 854.2 eV is neither found for Ni­(OH)_2_ samples nor for NiOOH.^68^ The signal at 855.9 eV corresponds to surface hydroxide and is typical of Ni 2p spectra of NiO.^68,69^

**Figure 6:**
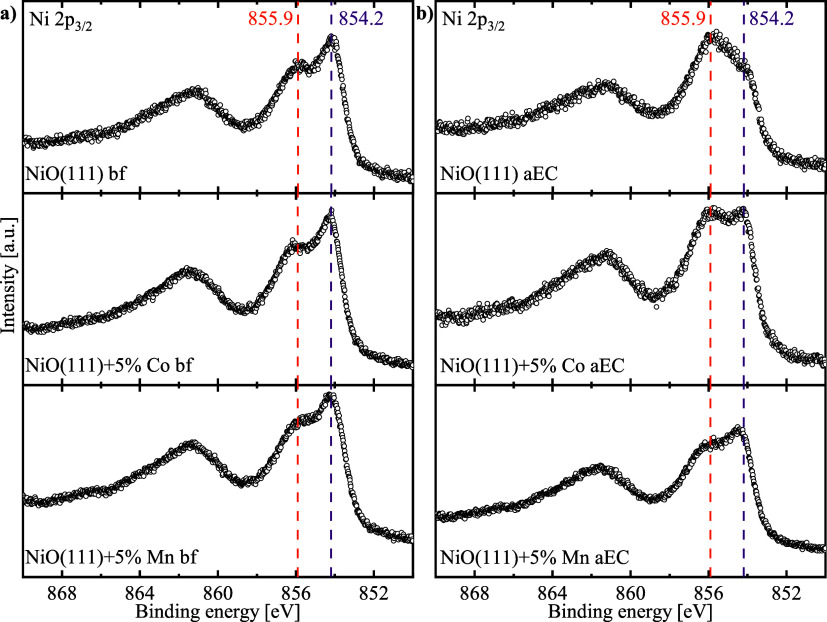
XPS spectra of (a) the Ni 2p_3/2_ of the NiO(111) samples without dopants, as well as with 5% Mn and Co doping (a) before electrochemistry (bf) and (b) after electrochemistry (aEC) with a stop potential of 1.7 vs RHE. All samples were measured as thin films on a GC substrate.

After electrochemistry, the survey spectra of the samples in Figures S22–24 reveal additional signals from K on the sample surface, originating from the KOH electrolyte that was not fully removed by rinsing the GC disc after the electrochemical experiment with ultrapure water. Figure 6b shows the Ni 2p_3/2_ spectra for the aEC-samples. For all samples, the feature at 855.9 eV became more dominant, but the magnitude of the peak change is different, with the NiO(111) aEC sample changing the most, followed by the 5% Co aEC sample and the 5% Mn sample. The feature at 855.9 eV indicates the partial formation of NiOOH and Ni­(OH)_2_ with the remaining NiO characterized by the decreased peak at 854.2 eV.^68^ This formation of NiOOH and Ni­(OH)_2_ from NiO after electrochemical treatment was reported previously for chemical vapor deposited NiO_*x*_ films as well as for thin film catalysts from hydrothermal synthesis.^46,70,71^ However, the decomposition of NiOOH to Ni­(OH)_2_ should be taken into account for ex situ experiments as recently reported.^72^

The Co 2p spectra as well as the Mn 2p spectra are shown in Figure S25. The spectral intensities were weak, which was explained by the low amount of dopant in the materials. The satellite at 786 eV in the Co 2p_3/2_ spectra indicates the contribution of a Co^II^ species, and the absence of a satellite in the Mn 2p spectra at 647 eV indicates that there are no Mn^II^ species.^69^ This suggests that the Co surface species in the bf material contain Co^II^ species and that the Mn surface species are predominantly Mn^III^ or higher. Both support the observation of the XAS results and indicate a similar surface composition to the bulk. The spacing between the doublets of the Mn 3s spectra, which is correlated with the Mn oxidation state, could not be fully resolved in this study.^73^

Figure 7a shows the corresponding O 1s spectra. The feature A at 529.8 eV is attributed to lattice oxide oxygen in NiO^69^ and is dominant in the bf-samples. The feature B is attributed to hydroxide oxygen or nonstoichiometric oxygen^69^ and feature C is attributed to adsorbed water and organic oxygen.^69^ Latter can be explained by the contribution of the sulfonic acid and fluorinated ether groups due to the Nafion content in the RDE films.^74,75^ The effect of Nafion is also observed in the high binding energy C 1s signals in the spectra, Figures S26 and S27. Comparing the different bf samples, the O 1s spectra are similar. In detail, the peak area ratio of features A/B/C in the O 1s spectra differs for the 5% Mn bf sample with 1/0.22/0.1 compared to those of the bf-NiO(111) and the 5% Co bf-samples with 1/0.27/0.1 and 1/0.26/0.1, respectively. The higher ratio of feature A in 5% Mn bf-sample could result from impurities of higher valent Mn phases (e.g., MnO_2_).

**Figure 7:**
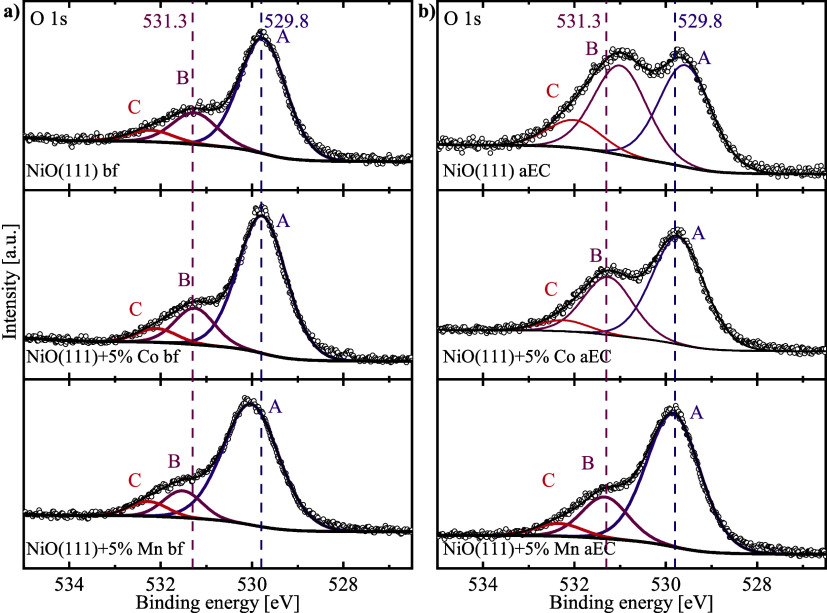
XPS spectra of the O 1s of the NiO(111) samples without dopants, as well as with 5% Mn and Co doping (a) before electrochemistry (bf) and (b) after electrochemistry (aEC) with a stop potential of 1.7 vs RHE. All samples were measured as thin films on a GC substrate.

Figure 7b shows the corresponding O 1s of the aEC samples. For all aEC samples, feature B became more dominant, together with the lower relative amount of feature A, supporting the hypothesis of NiOOH and Ni­(OH)_2_ formation on the surface of the materials after electrochemistry.^71^ It is also likely that some residual KOH contributes to feature B, but based on the relatively low amounts of observed K in the survey, this is a rather negligible contribution. As for the Ni 2p_3/2_ spectra, the O 1s spectra of the aEC samples also have a different magnitude of change depending on the dopant. The 5% Mn-doped sample shows the least change in the relative peak area of feature B. In fact, the relative ratio of B changes from 0.22 to 0.3 for the 5% Mn from bf to aEC samples. The relative ratio of feature B of the 5% Co aEC sample changes from 0.26 to 0.54. The feature B of pure NiO(111), on the other hand, has the largest change of feature B from 0.27 to 0.88. These observations indicate that the surface of pure NiO(111) transforms the most to a NiOOH/Ni­(OH)_2_-rich surface layer in agreement with oxidation changes from the XANES analysis and oxidation charges of the cyclic voltammetry data. In contrast, doping with Co and Mn hinders the strong transformation of the NiO host structure to a NiOOH/Ni­(OH)_2_ with Mn as the most preventive dopant. The latter agrees with a minor change in XANES data. Interestingly, the relative contributions and changes of the discussed XPS features also correlate with CV analysis, where a strong redox peak was observed for pure NiO(111), indicating a significant reconstruction of the surface with (oxy)­hydroxide formation. Lower redox charges were observed for the doped samples. The modest transformation of Co- and Mn-doped samples to the catalytically active NiOOH-terminated surface might explain the low activity increase by electrochemical activation in OER evaluation. Because the addition Co and Mn is expected to improve the OER electrode activity, we interpret the findings in the way that the decreased NiOOH formation and for the Mn doping also the leaching of the Mn minimize the effect of the activity improvement, whereas for the case of Mn-doping, the OER electrode activity was even decreased.

## 4 Conclusions

In this study, Co- and Mn-doped NiO nanosheets with a preferential (111) faceted surface structure were synthesized by a MW-assisted synthesis route with a focus on low doping levels between 2 and 10 mol %. In this regime, doping was shown to be effective in maintaining the crystallinity of the rock salt NiO host structure and phase-purity. Thus, the MW-assisted synthesis successfully enabled the preparation of a morphology-controlled, doped Ni-oxide catalyst. While the crystal structure and morphology remained more or less identical, an influence on the physical BET surface area was observed. Interestingly, Mn doping resulted consistently in an increase of the BET surface area compared to the pure NiO(111) sample, which might be explained by folded morphology and smaller pore sizes visible in TEM measurements. In contrast, Co doping with a doping level of 10% and higher resulted in lower BET surface areas.

The trends in the specific surface area were partially contrary to the electrochemical activity trends, where higher oxidation charges in cyclic voltammetry measurements were observed for the pure NiO(111) sample in comparison to 5% Co and Mn doped samples. The effect of Co and Mn dopants and different doping levels on the OER electrode activity was modest compared to the generally reported activity enhancement of bimetallic transition metal oxides. In case of Mn doping, the OER overpotential even increased from 481 mV of the pure NiO(111) to 537 mV of the NiO(111) + 10% Mn sample. This behavior was explained by a different extent of surface reconstruction of the rock salt precatalyst to a NiOOH-terminated surface in the case of doping. The hypothesis of the differences in surface reconstruction was analyzed with XPS investigations of the catalysts before and after electrochemical tests. XPS revealed a modest change of Mn-doped samples relating to the lesser degree of hydroxide formation. The minor extent of surface reconstruction of oxide precatalysts into the hydroxides of the Mn-doped samples may hinder the formation of a more active NiOOH surface during OER and thus limit the OER activity. Furthermore, the instability of the Mn material should be the focus of future studies. The Co-doped samples also exhibited a low surface reconstruction while decreasing the OER overpotential for lower doping levels (<10 mol %). The OER overpotential increase at higher doping levels (<10 mol %) was attributed to the hindered reconstruction by Co_3_O_4_ impurities. Thus, electrochemical cycling seems not to be an effective strategy for provoking surface reconstruction of higher Co-doped materials.

The XAS analysis is in accordance with the electrochemical results, indicating a lack of changes in the bulk Ni oxidation state with doping, together with modest changes in activity with doping. The absolute oxidation states of Ni are close to 2+, which is among the least active for OER. Co^II^ and Mn^III^/Mn^IV^ likewise are not very active, and the doping of Mn even leads to Mn dissolution. Generation-collection experiments of Mn dissolution by RRDE suggest only low corrosion of Mn from the film and can partially explain the low OER electrode activity and the minor degree of hydroxide formation. The main driver for activity may be different factors, namely, dopant metal sites in an untypical bonding environment, if the site persists on the surface, the formation of surface hydroxides, and changes in roughness.

Overall, the results showed the combination of facet-control and transition metal doping for Ni oxide-based catalysts as a promising strategy to study the effect of Co and Mn doping on the OER activity. However, further material optimization by tuning synthesis parameters will be necessary to develop more active catalysts, for example, by a successful incorporation of Fe into the faceted materials.

## Supplementary Material



## Notes

Supplementary schematics, electrochemical protocol, tabulated ICP-MS results, supporting figures from physical, electrochemical, and spectroscopic characterization, and different activity metrics (PDF)
