# Supplementary material for: Influence of Co and Mn Doping on the Surface Reconstruction of Faceted NiO(111) Nanosheets after the Oxygen Evolution Reaction
Source: J Phys Chem C Nanomater Interfaces. 2025 May 10;129(20):9341–55. doi: 10.1021/acs.jpcc.5c00493 (PMC12105031; doi:10.1021/acs.jpcc.5c00493)
Supplement: Supplementary file 1 [file jp5c00493_si_001.pdf]

Supporting Information

Influence of Co and Mn Doping on the Surface Reconstruction of Faceted NiO(111) Nanosheets After Oxygen Evolution Reaction

Konstantin K. Rücker[a,b], Dereje Hailu Taffa[b], Omeshwari Bisen[c], Marcel Risch[c], Darius Hayes[d], Elliot Brim[d], Ryan M. Richards[d,e], Corinna Harms[a], Michael Wark[b], Julian Lorenz[a]**

[a] Institute of Engineering Thermodynamics, German Aerospace Center (DLR), Carl-von-Ossietzky-Str.15, 26129 Oldenburg, Germany.

[b] Institute of Chemistry, Chemical Technology I, Carl von Ossietzky University of Oldenburg, Carl-von-Ossietzky-Str. 9-11, 26129 Oldenburg, Germany.

[c] Nachwuchsgruppe Gestaltung des Sauerstoffentwicklungsmechanismus, Helmholtz-Zentrum Berlin für Materialien und Energie GmbH, Hahn-Meitner-Platz 1, 14109 Berlin, Germany.

[d] Department of Chemistry, Colorado School of Mines, 1500 Illinois St., Golden, Colorado 80401.

[e] Chemical and Material Sciences Center, National Renewable Energy Laboratory, Golden, Colorado 80401.

**konstantin.ruecker@dlr.de*

**julian.lorenz@dlr.de*

**marcel.risch@helmholtz-berlin.de*

Table of Contents

Supporting information S3
S1. Schematic nanosheet synthesis S3
S2. Schematic XAS set-up S4
S3. Measurement sequence for electrochemical characterization S5
S4. ICP-MS analysis S6
S5. PXRD of higher Co doped samples S6
S6. Energy dispersive x-ray spectroscopy S7
S7. BET Isotherms S8
S8. Additional EC Characterization of Cycling S8
S9. ICP-MS of the electrolyte for determination of Fe S9
S10. Generation Collection Experiments S10
S11. Cyclovoltammetrie of each replicate experiment for OER electrode activity determination S13
S12. Electrode activities of higher doped Co samples S17
S13. Results for the estimation of the double layer capacitance S18
S14. Different metrics for the electrocatalytic electrode activities S21
S15. XAS of every doping level S22
S16. EXAFS of different Mn k edge reference Materials S23
S17. XPS Survey spectra S24
S18. XPS Co and Mn 2p spectra S26
S19. XPS C 1s spectra S27
References S28

Supporting information

S1. Schematic nanosheet synthesis

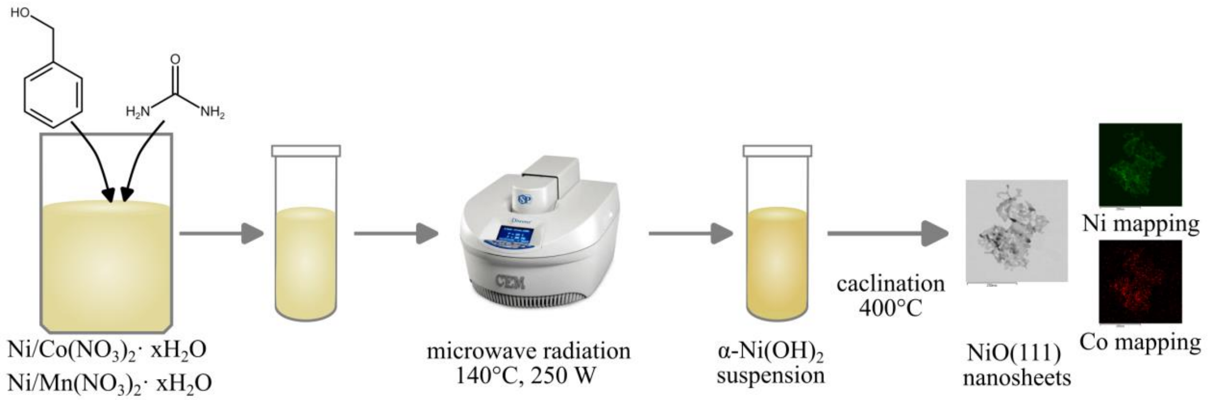

Figure S1: Schematic of the synthesis of doped transition metal oxides by microwave-assisted precipitation of hydroxides and annealing at 400°C to obtain NiO(111) nanosheets. The EDS mapping for the Example of Co doping is shown.

S2. Schematic XAS set-up

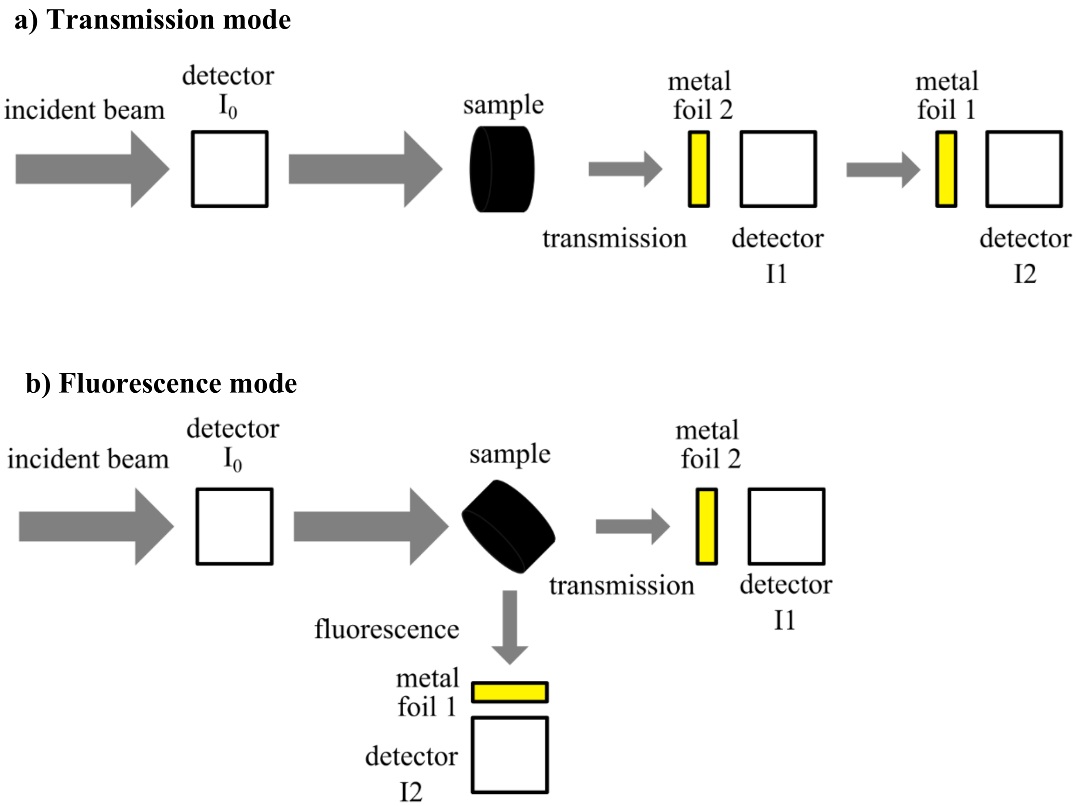

Figure S2: Schematic of the X-ray absorption spectroscopy experiments at KMC-2. The detector I_0 measures the incident intensity. a) In transmission mode, the sample is placed straight in the beam path and between the detector I1 and the sample, metal foil 1 of the edge with the next lower energy (e.g. Cr foil for Mn edge, Fe foil for Co edge and Co foil for Ni edge) was placed as a filter. The corresponding metal for referencing the energy edge is placed in front of detector I2 as metal foil 2. b) In the case of the fluorescence mode, the sample is placed within a 45° angle between the beam path and the detector I2 with metal foil 1 as a filter.

S3. Measurement sequence for electrochemical characterization

Table S1: Steps of the electrochemical characterization.

**Step**	**Conditions**
**1. Cleaning**	Polish and clean the electrodes. The cell is sonicated multiple times in pure water.
**2. Nitrogen purge at OCP**	The electrolyte is purged while the coated electrode is rested at OCP in the electrolyte for 30 min.
**3. EIS in Nitrogen**	Frequency: 30 kHz-1 Hz<br>Points/decade: 10<br>10 mV amplitude at 1.0 V vs. RHE
**4. LSV for CDL**	Hold for 20 s at 0.9 V vs. RHE<br>Potential window: 0.9 - 1.1 V vs. RHE<br>Scan rates: 5, 10, 25, 50, 100, 250, 500 mV s^{-1}<br>Hold for 20 s at 1.1 V vs. RHE<br>Repeat for backward scan 1.1 – 0.9 V vs. RHE<br>Rotation: static<br>No dynamic iR compensation.
**5. Oxygen purge at OCP**	15 min
**6. EIS in Oxygen before cycling**	Frequency: 30 kHz-1 Hz<br>Points/decade: 10<br>10 mV amplitude at 1.0 V vs. RHE
**7. CV for OER initial**	Hold for 120 s at start potential 1.1 V vs. RHE<br>Potential window: 1.0 – 2.0 V vs. RHE<br>Scan rates: 10 mV s^{-1}<br>Scans: 3<br>Rotation: 2400 rpm<br>No dynamic iR compensation.
**8. CV for EC cycling**	Hold for 20 s at start potential 1 V vs. RHE<br>Potential window: 1.0 – 1.7 V vs. RHE<br>Scan rates: 100 mV s^{-1}<br>Scans: 350<br>Rotation: 1600 rpm<br>No dynamic iR compensation.
**9. CV for OER cycled**	Hold for 120 s at start potential 1.1 V vs. RHE<br>Potential window: 1.0 – 2.0 V vs. RHE<br>Scan rates: 10 mV s^{-1}<br>Scans: 3<br>Rotation: 2400 rpm<br>No dynamic iR compensation.
**10. EIS in Oxygen after cycling**	Frequency: 30 kHz-1 Hz<br>Points/decade: 10<br>10 mV amplitude at 1.0 V vs. RHE

S4. ICP-MS analysis

Table S2: Table of the ICP-MS results of the metal oxides after dissolution in concentrated nitric acid given as molar ratio of the metal content.

Sample	x_{Co} / mol %	x_{Mn} / mol %	x_{Ni} / mol %
NiO(111)	0.1	0.0	100.1
NiO(111)+2%Co	2.3	-	97.8
NiO(111)+5%Co	5.1	-	95.2
NiO(111)+10%Co	10.2	-	90.0
NiO(111)+20%Co	18.9	-	81.1
NiO(111)+40%Co	33.0	-	67.0
NiO(111)+60%Co	46.4	-	53.6
NiO(111)+2%Mn	-	2.3	97.0
NiO(111)+5%Mn	-	5.0	95.1
NiO(111)+10%Mn_	-	10.1	90.1

S5. PXRD of higher Co doped samples

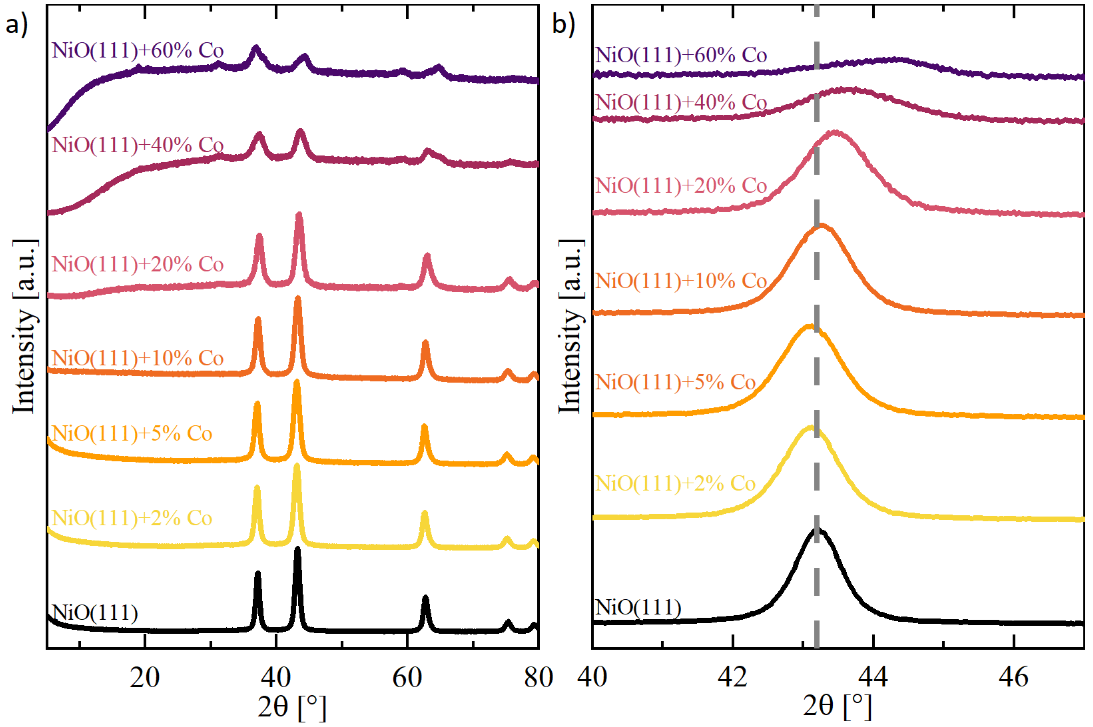

Figure S3: a) PXRD of cobalt doped Nickel oxide (111) nanosheets with doping up to 60% in which impurities of Co_3O_4 occur at doping levels of 20% and higher. b) Graph of a zoom on the (002) signal.

S6. Energy dispersive x-ray spectroscopy

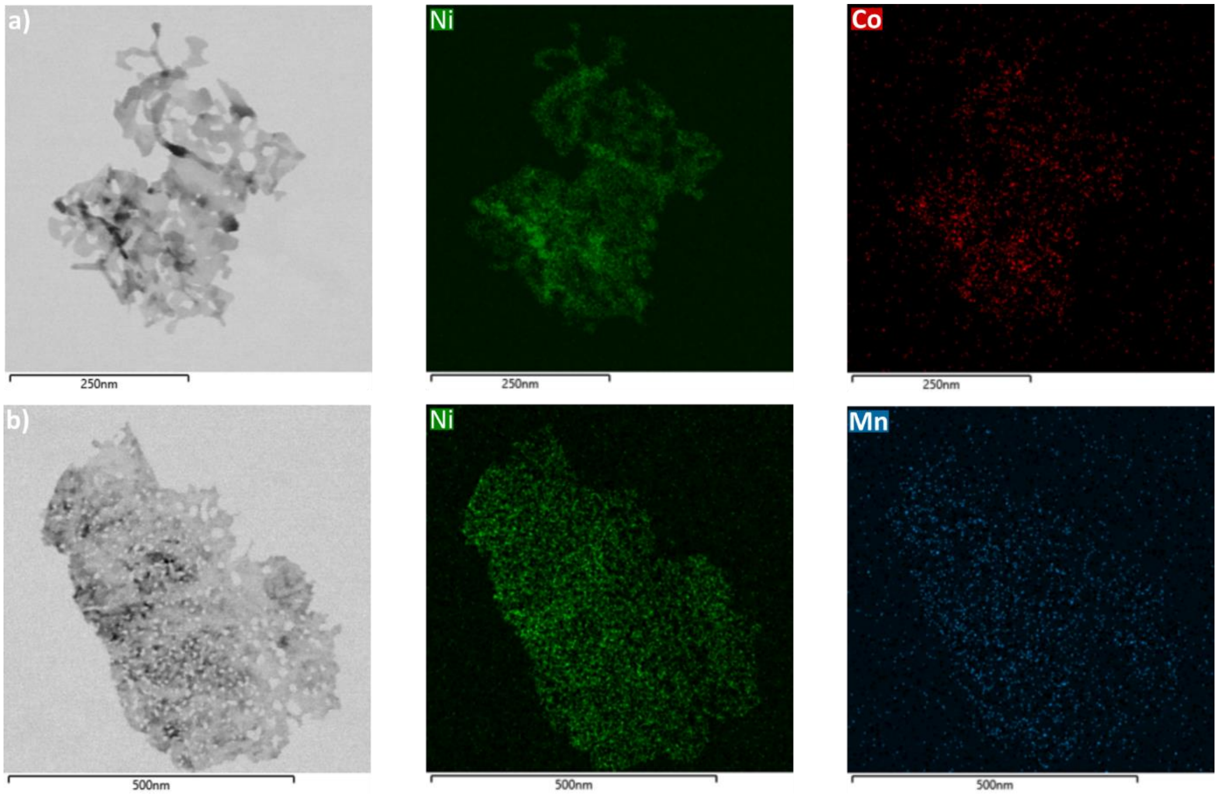

Figure S4: TEM images and EDS elemental mapping of a) The NiO(111)+5% Co and b) NiO(111)+5% Mn.

S7. BET Isotherms

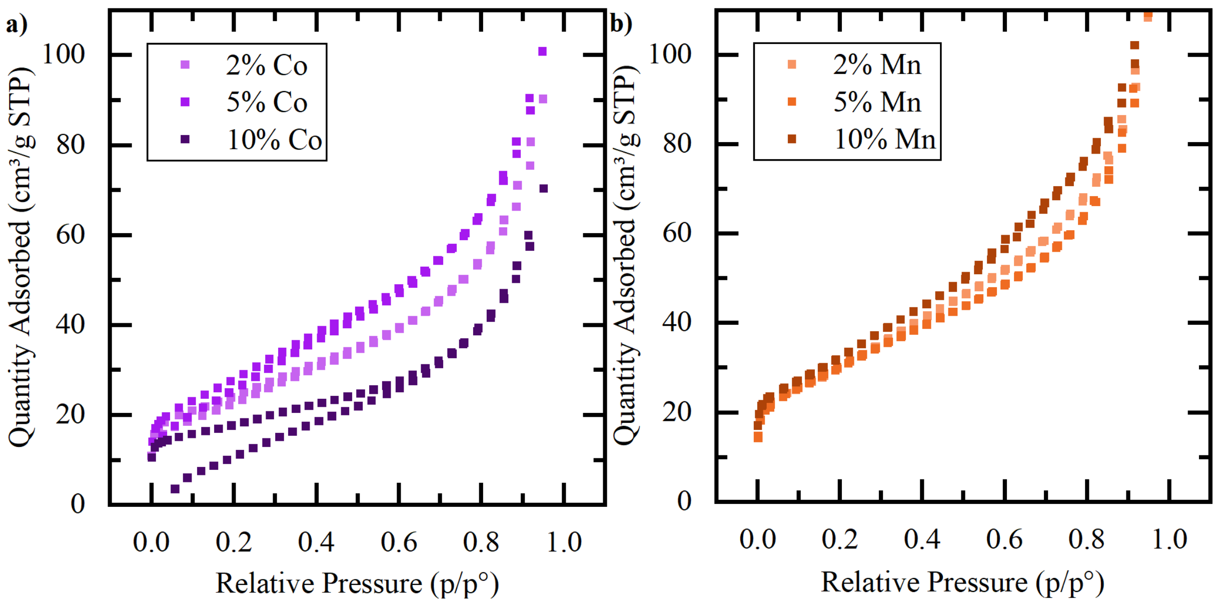

Figure S5: Nitrogen absorption-desorption isotherms of a) the cobalt doped and b) the manganese doped samples.

S8. Additional EC Characterization of Cycling

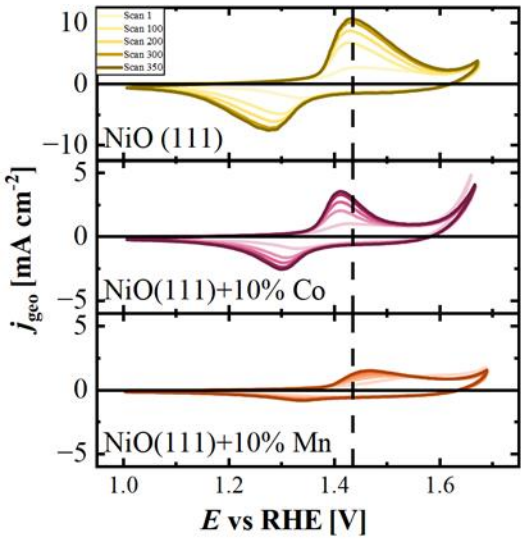

Figure S6:Cyclovoltammogram of the Scan 1, 100, 200, 300 and 350 of the 10% doped Co and Mn sample in comparison to the pure NiO sample.

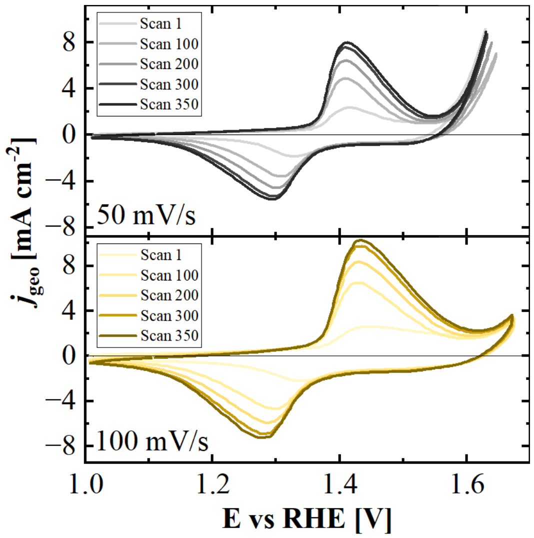

Figure S7: Cyclic voltammograms of the Scan 1, 100, 200, 300 and 350 of the NiO(111) sample with different scan rates of 50 mV/s and 100 mV/s. The currents of the Ni^{II}/Ni^{III} oxidations are smaller with slower scan rate, and the apparent OER onset currents are larger. This implies a more effective activation of the pre-catalyst at slower scan rates. We note that slower scan rate with the same number of scans result in longer durations at respective potentials during activation.

S9. ICP-MS of the electrolyte for determination of Fe

Table S3: ICP-MS results of the 0.1 M KOH solution used in the experiments. The measurements were performed as three independend experiments. Note that the detection limit of Fe was about 0.5 µg L^{-1} because of interferences of ^{56}Fe with the ArO^+ from the plasm and carrier gas Ar. However, the 1.5 µg L^{-1} was sufficiently resolved.

**Element**	**Mn**	**Fe**	**Ni**	**Co**
**Middle value [µg L^{-1}]**	0.053	1.5	0.06	0.048
**Standard deviation [µg L^{-1}]**	0.003	0.3	0.01	0.008

S10. Generation Collection Experiments

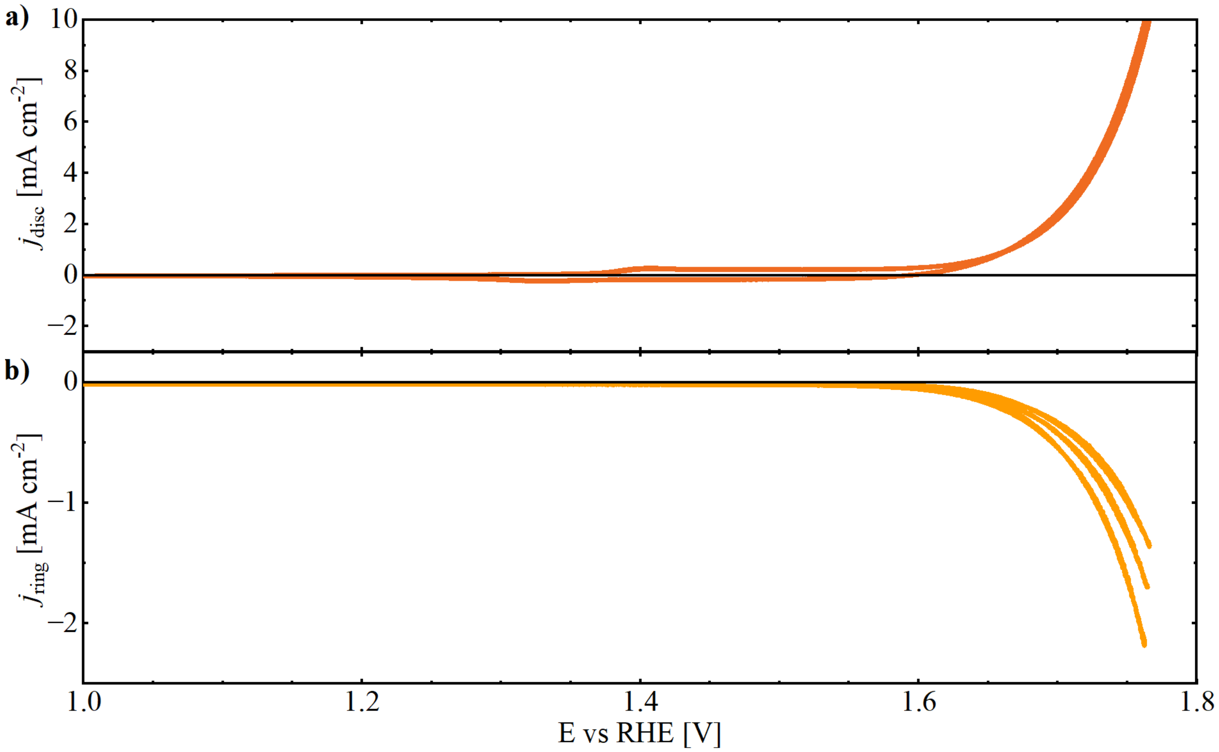

Figure S8: Cyclic voltammograms of a) the disc current and b) collector experiment of the corresponding current of the Platinum ring with a ring potential set at 0.4 V vs. RHE for detection of O_2 generated during OER with an onset at approximately 1.55 V vs. RHE.[1] The experiment was performed with a 100 µg cm^{-2} loading of NiO(111)+10% Mn Material in N_2-saturated 0.1 M KOH solution. The Pt ring was cycled by 100 CVs between 0 and 1.1 V vs. RHE at 100 mV s^{-1} previous to the experiment as described elsewhere.[2]

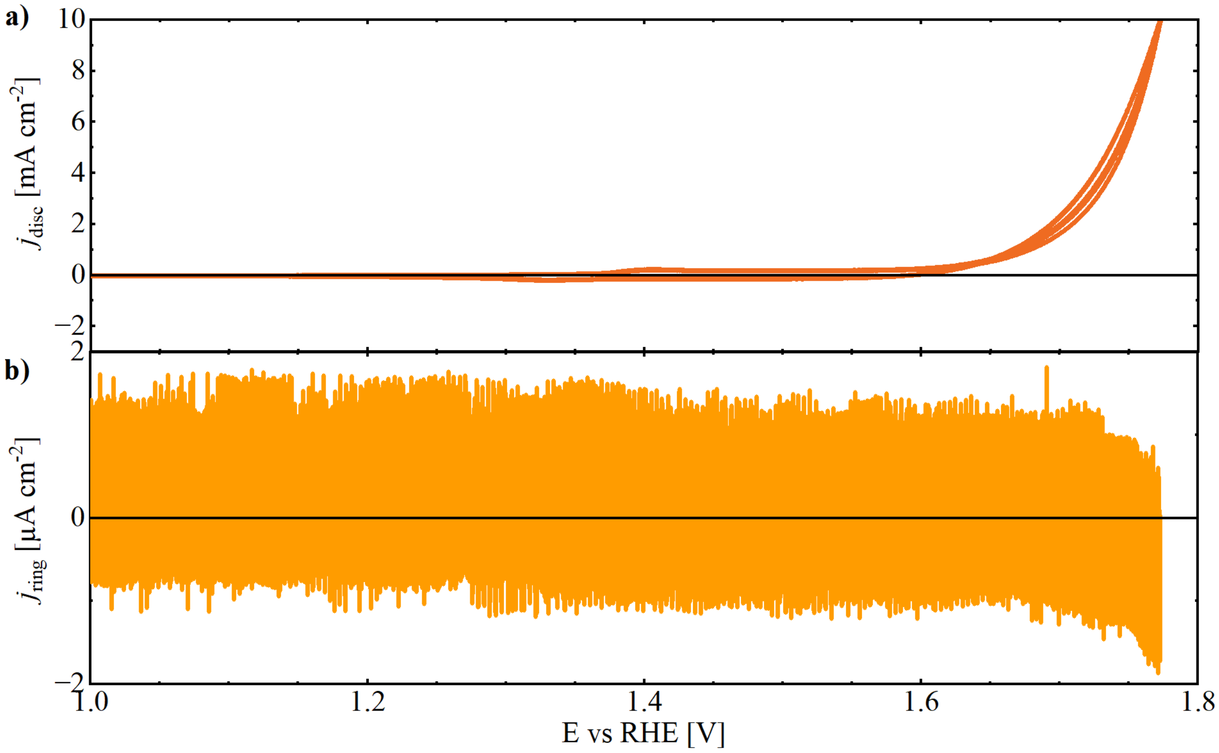

Figure S9: Cyclic voltammograms of a) the disc current and b) collector experiment of the corresponding current of the Platinum ring with a ring potential at 1.2 V vs. RHE for the detection of Mn corrosion current. The experiment was performed with a 100 µg cm-2 loading of NiO(111)+10% Mn Material in N_2-saturated 0.1 M KOH solution. The Pt ring was cycled by 100 CVs between 0 and 1.1 V vs. RHE at 100 mV s^{-1} previous to the experiment as described elsewhere.[2] The ring currents were low and noisy but a reduction current was observed at disc potentials above 1.7 V vs. RHE

Table S4: ICP-MS results of the generation-collection experiments in 0.1 M KOH electrolyte from Experiment S7-S8 and calculation of the approximate fraction of dissolved catalyst from the RDE catalyst layer assuming the stoichiometry $Ni_9Mn_1O_{10}$

**c(Mn, electrolyte)**	0.902 μg L^{-1}
**c(Ni, electrolyte)**	0.719 μg L^{-1}
**V(electrolyte)**	0.11 L
**m(Mn, electrolyte)**	99.2 ng
**m(Ni, electrolyte)**	79.1 ng
**m(NiO(111)+10% Mn, electrode)**	19.6 μg
**m(Mn, electrode, assuming $Ni_9Mn_1O_{10}$)**	1.5 μg
**m(Ni, electrode, assuming $Ni_9Mn_1O_{10}$)**	14 μg
**ω (Mn, from electrode in electrolyte)**	7%
**ω (Ni, from electrode in electrolyte)**	0.6%

S11. Cyclovoltammetrie of each replicate experiment for OER electrode activity determination

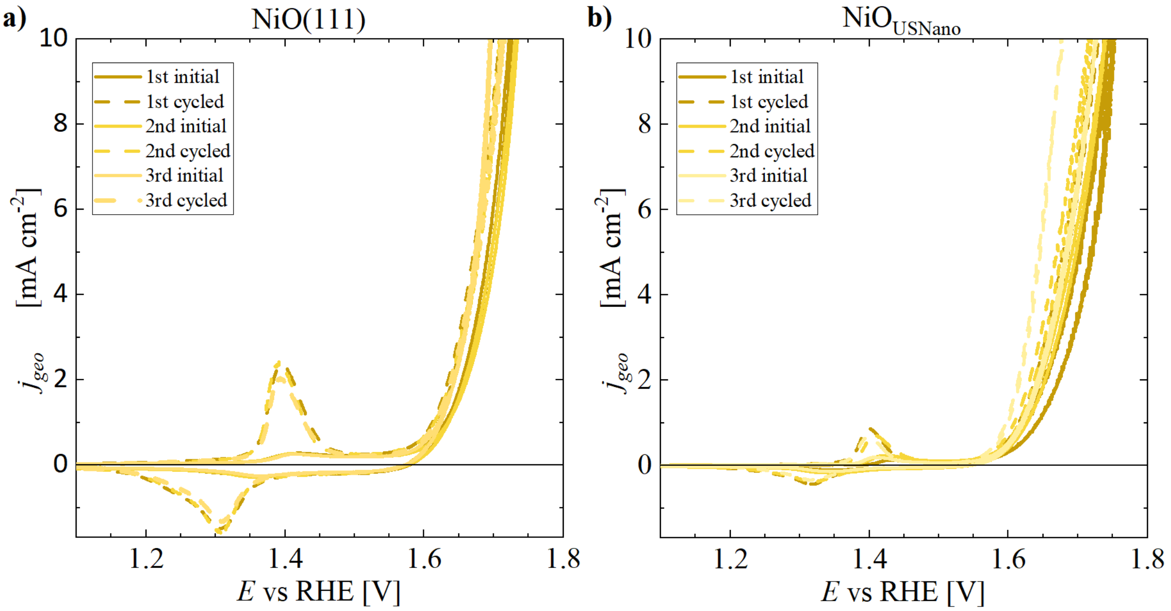

Figure S10: Cyclic voltammograms of a) pure NiO(111) and b) NiO commercial. All CVs represent the results of the independent electrodes and are the third scans at 10 mV s^{-1} initial and cycled (after 350 CV).

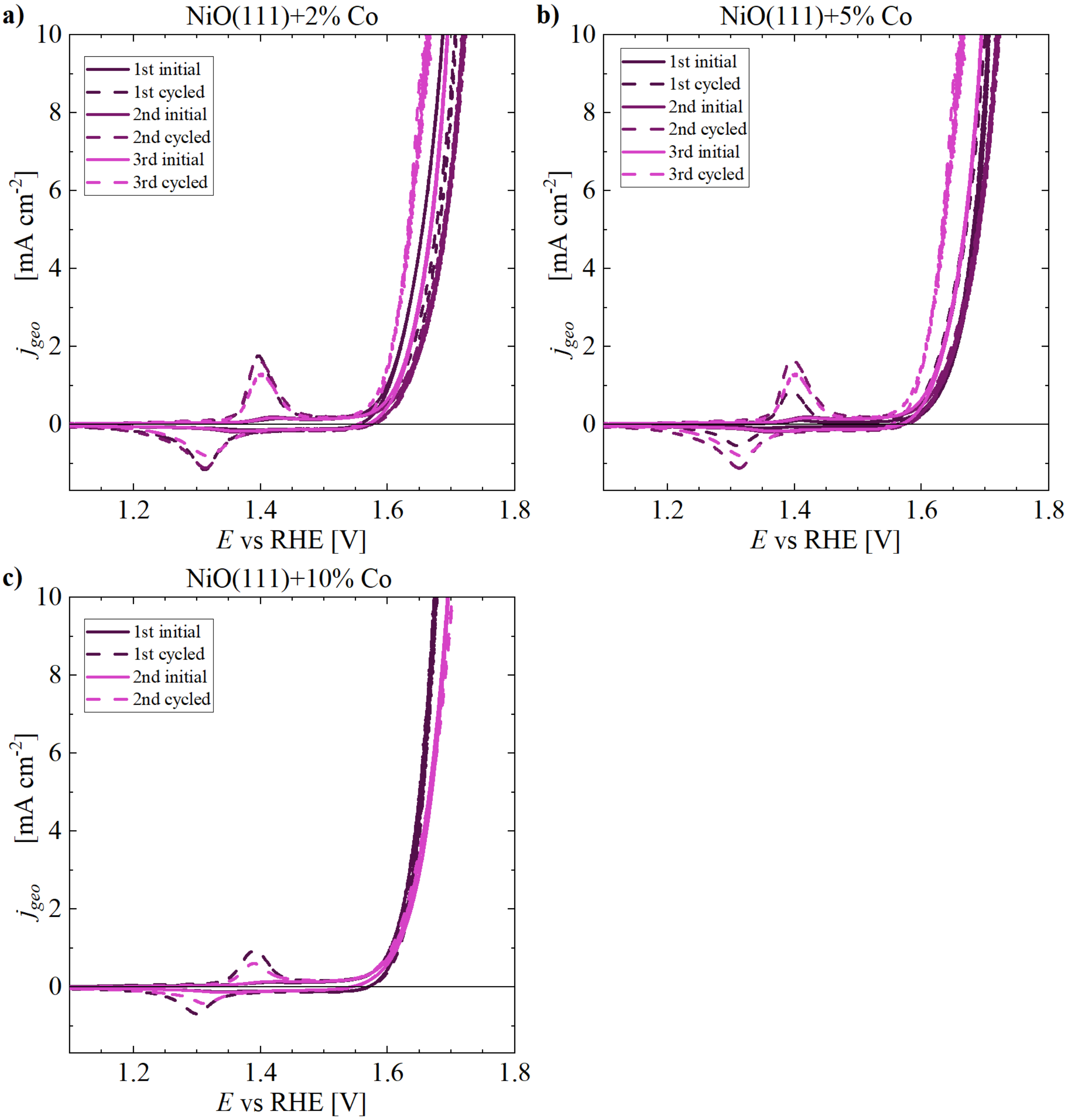

Figure S11: Cyclic voltammograms of NiO(111) samples with a) 2%, b) 5% and c) 10% Co doping. All CVs represent the results of the independent electrodes and are the third scans at 10 mV s^{-1} initial and cycled (after 350 CV).

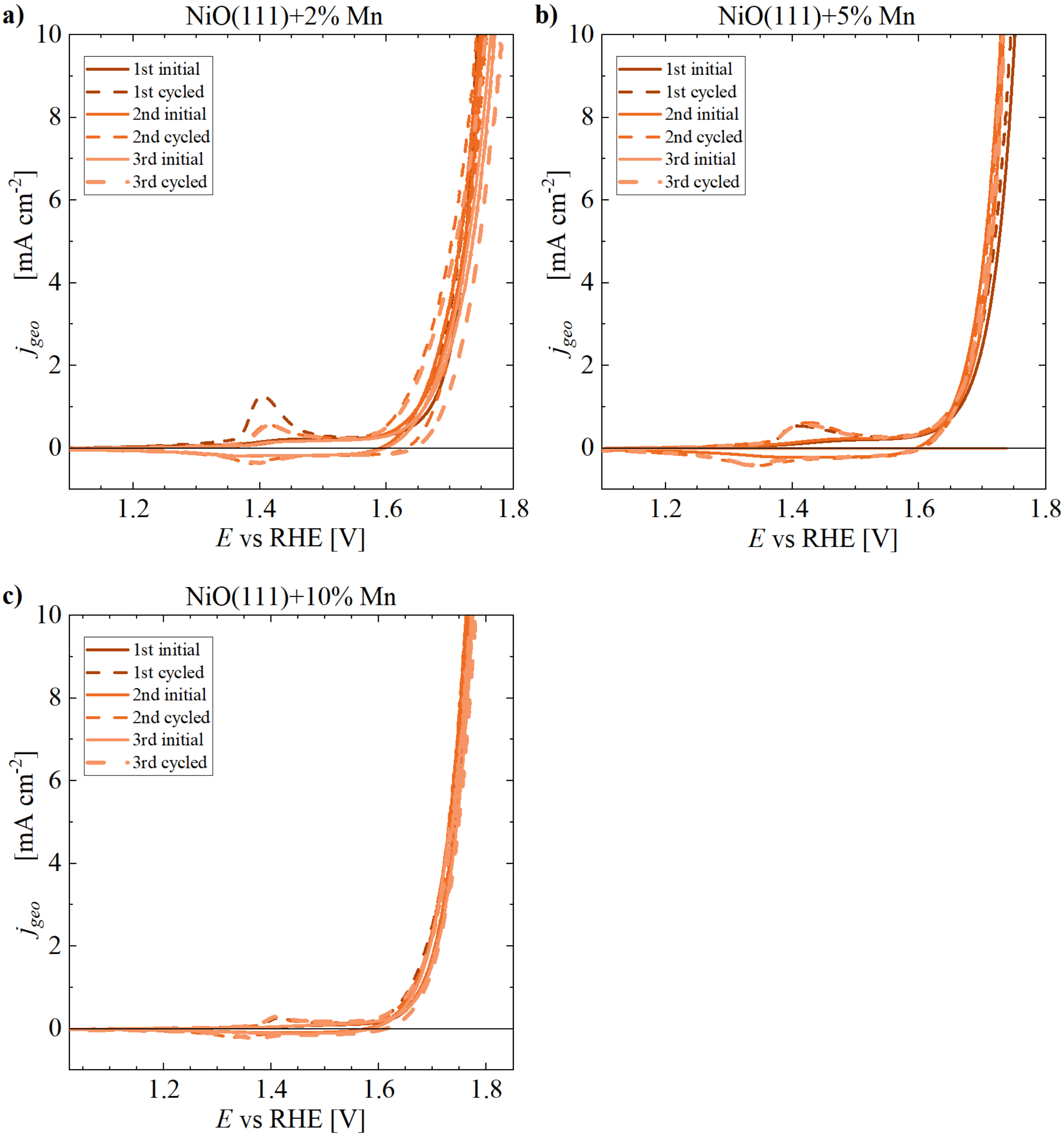

Figure S12: Cyclic voltammograms of NiO(111) samples with a) 2%, b) 5% and c) 10% Mn doping. All CVs represent the results of the independent electrodes and are the third scans at 10 mV s^{-1} initial and cycled (after 350 CV).

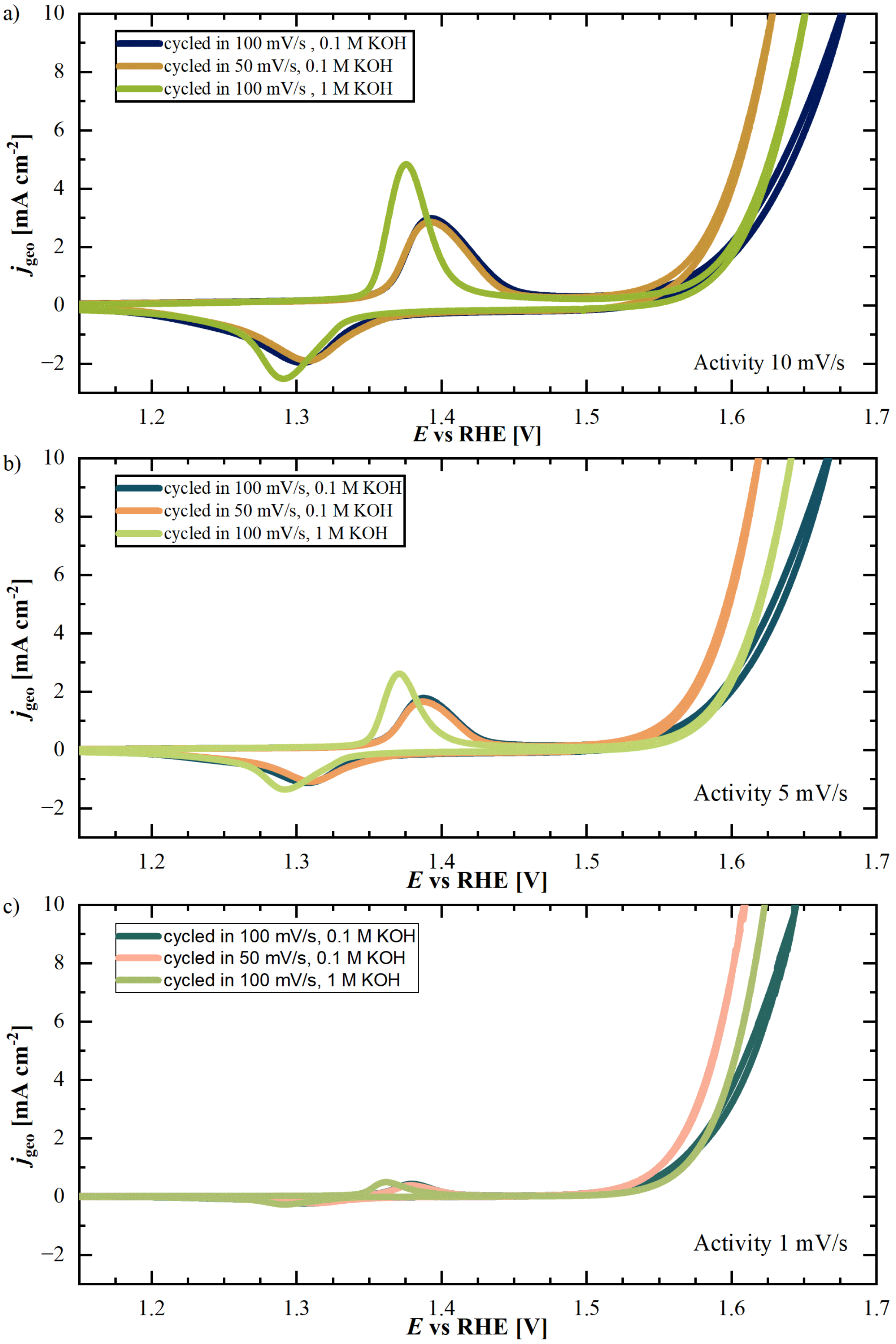

Figure S13: Cyclic voltammograms of the NiO(111) sample at different activation conditions. The cyclic voltammograms were measured after cycling of the catalyst films for 350 cycles with different scan rates as well as electrolyte concentrations and were recorded with a) 10 mV/s b) 5 mV/s and c) 1 mV/s. The

characteristic Ni^{II}/Ni^{II} oxidation peak is sharper after cycling in the 1 M KOH. The material is more active after cycling with 50 mV/s in 0.1 M KOH, indicating the effectiveness of slower cycling over the electrolyte concentration.

S12. Electrode activities of higher doped Co samples

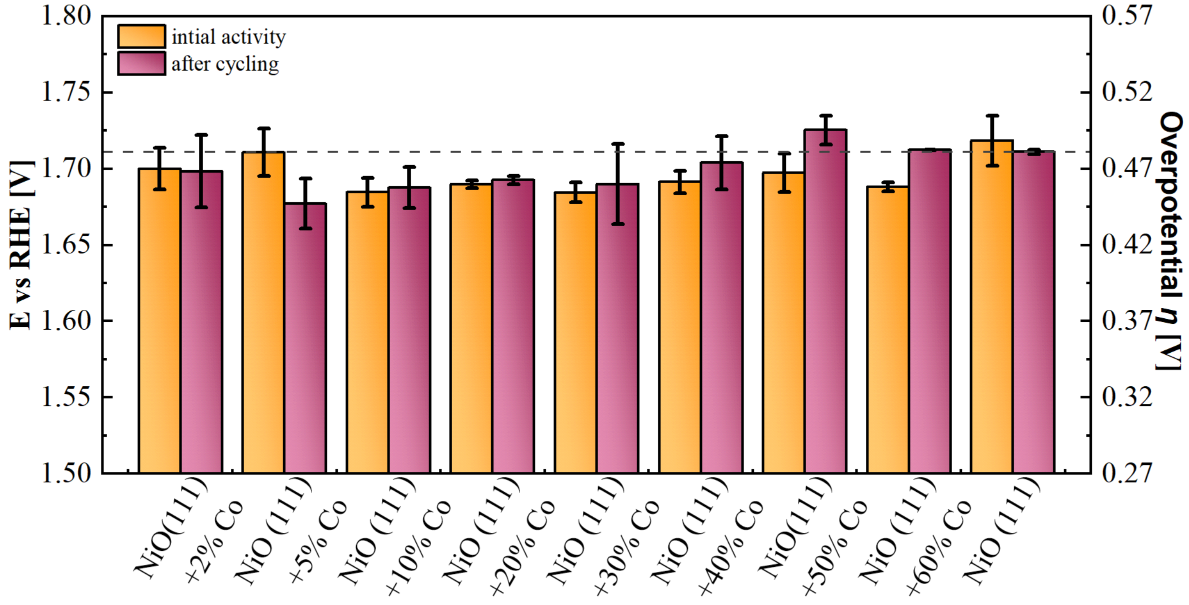

Figure S14: Overpotentials of higher doped Co samples at 10 mA cm-2 with a line at the overpotential of NiO(111) after cycling as guide to the eye.

S13. Results for the estimation of the double layer capacitance

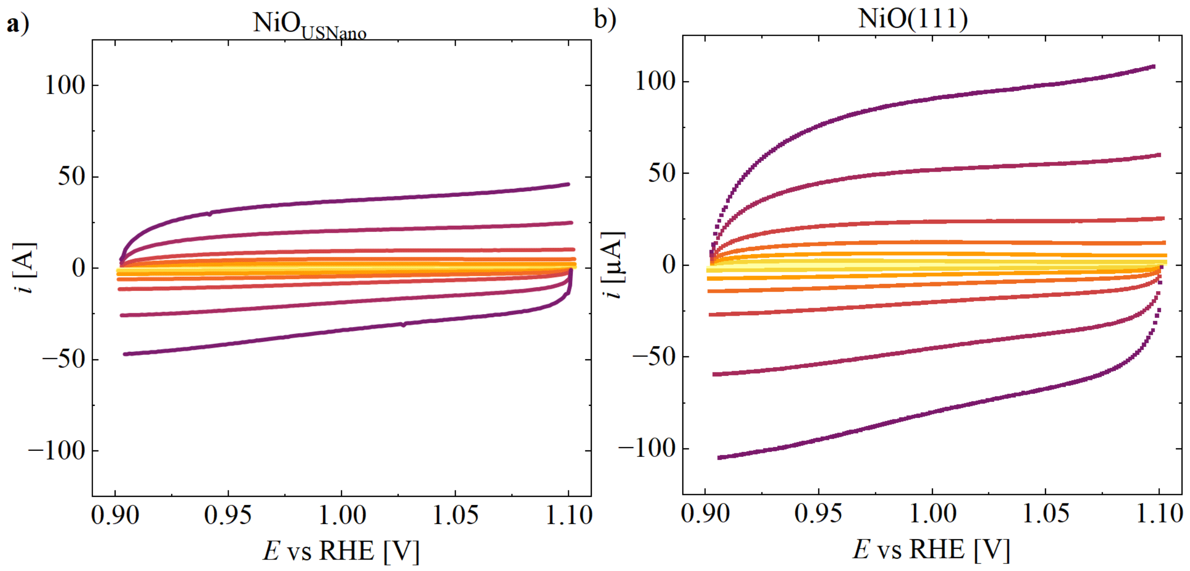

Figure S15: Anodic and cathodic linear sweep voltammetry with scan rates from 5 to 500 mV s-1 between 0.9 and 1.1 V vs. RHE of a) NiO_{USNano} commercial and b) NiO(111).

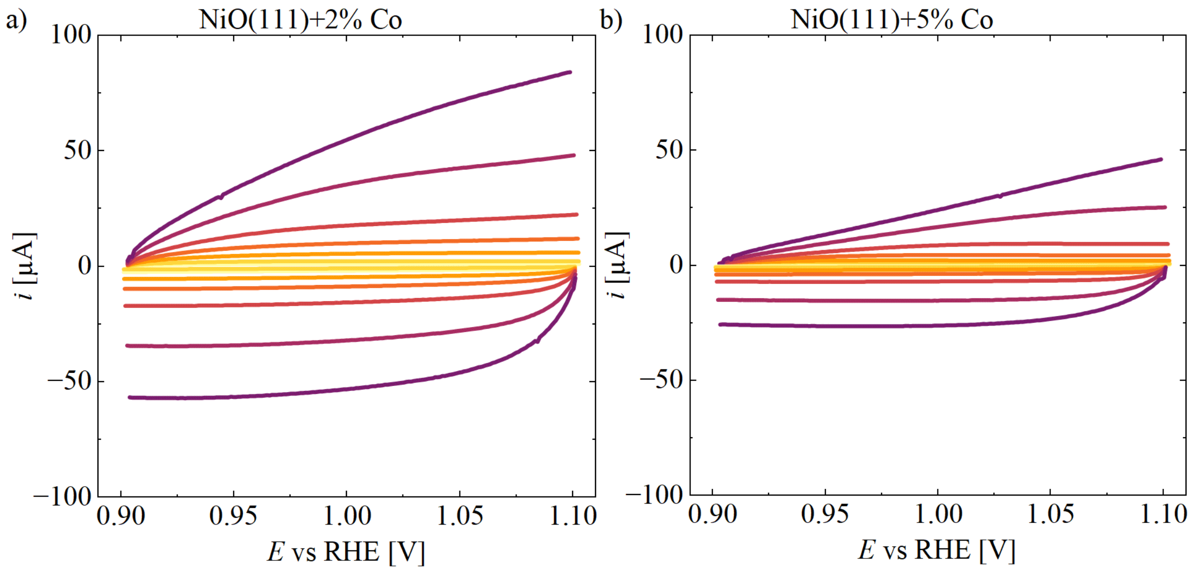

Figure S16: Anodic and cathodic linear sweep voltammetry with scan rates from 5 to 500 mV s-1 between 0.9 and 1.1 V vs. RHE of a) NiO(111) +2% Co and b) +5% Co.

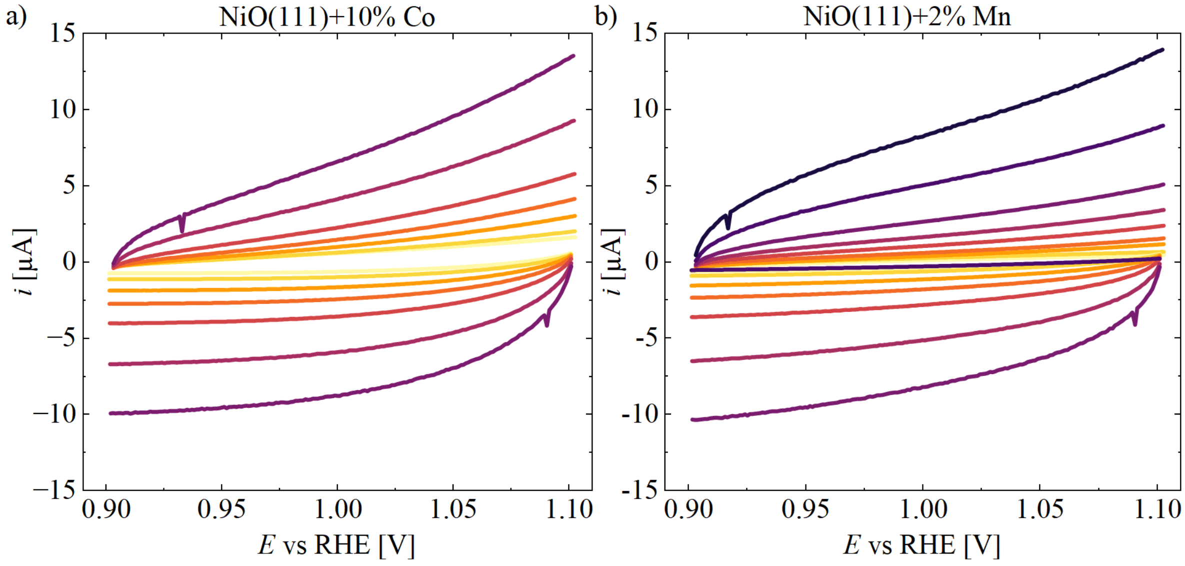

Figure S17: Anodic and cathodic linear sweep voltammetry with scan rates from 5 to 500 mV s-1 between 0.9 and 1.1 V vs. RHE of a) NiO(111) + 10% Co and b) + 2% Mn

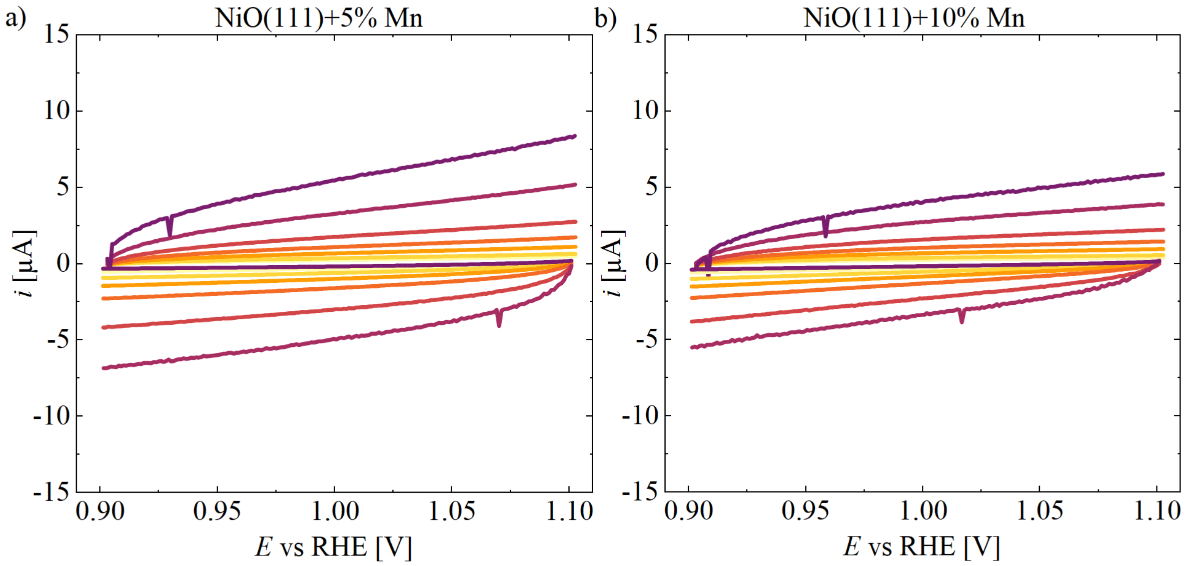

Figure S18: Anodic and cathodic linear sweep voltammetry with scan rates from 5 to 500 mV s-1 between 0.9 and 1.1 V vs. RHE of a) NiO(111) + 5% Mn and b) + 10% Mn.

Table S5: Parameters C_{DL} and α from the regression of the currents as a function of the scan rates from negative going lineare sweeps different materials. α-values different than 1 describe a deviation from an ideal capacitor.

**Sample**	**C_{DL} [mF cm^{-2}]**	**α**
NiO(111)	0.92	0.87
NiO_{Nano}	0.34	0.88
NiO(111)+2% Co	0.49	0.76
NiO(111)+5% Co	0.24	0.68
NiO(111)+10% Co	0.074	0.56
NiO(111)+2% Mn	0.031	0.64
NiO(111)+5% Mn	0.067	0.67
NiO(111)+10% Mn	0.045	0.56

S14. Different metrics for the electrocatalytic electrode activities

Table S6: Common electrode activity metrics for the electrochemically tested samples, including the overpotential at 10 mA cm_{geo}^{-2}, the current density normalized to the BET surface area at 1.65 V vs. RHE j_{BET} and the current density normalized to the capacitance at 1.65 V vs. RHE j_{CDL}.

**Sample**	**η at 10 mA cm^{-2} before cycling [mV]**	**η at 10 mA cm^{-2} after cycling [mV]**	**j_{BET} at 1.65 V [nA cm_{BET}^{2}]**	**j_{CDL} at 1.65 V [mA mF^{-1}]**
NiO(111)	488 ± 16	481 ± 1	40.2	3.09
NiO_{Nano}	508 ± 10	476 ± 22	142	11.8
NiO(111)+2% Co	470 ± 14	468 ± 24	36.4	7.86
NiO(111)+5% Co	481 ± 16	447 ± 16	38.3	16.8
NiO(111)+10% Co	455 ± 10	457 ± 14	34.8	53.7
NiO(111)+2% Mn	525 ± 11	507 ± 5	20.5	56.0
NiO(111)+5% Mn	510 ± 8	512 ± 3	10.7	13.7
NiO(111)+10% Mn	540 ± 4	537 ± 2	8.71	11.9

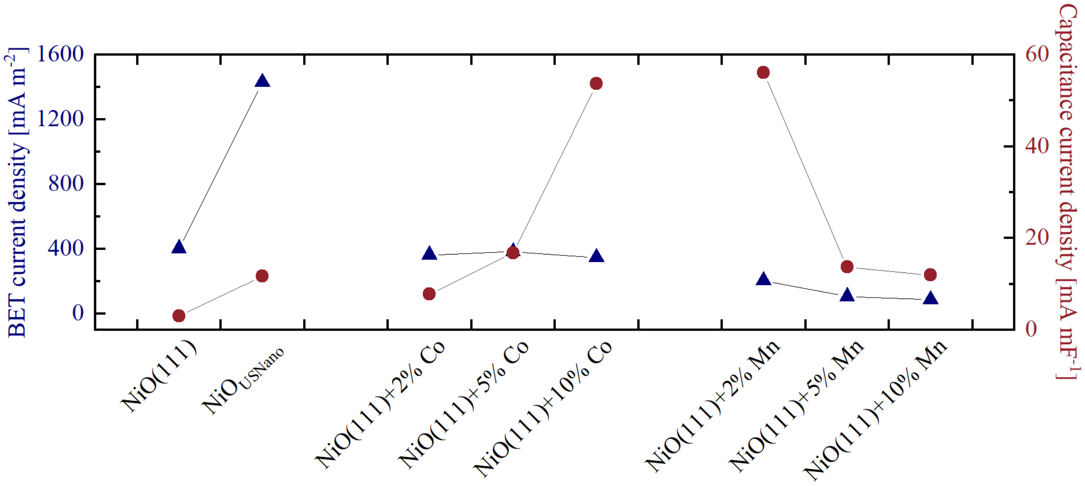

Figure S19: Plot of the activity metrics of the current density normalized to the BET surface area at 1.65 V vs. RHE and the current density normalized to the capacitance at 1.65 V vs. RHE.

S15. XAS of every doping level

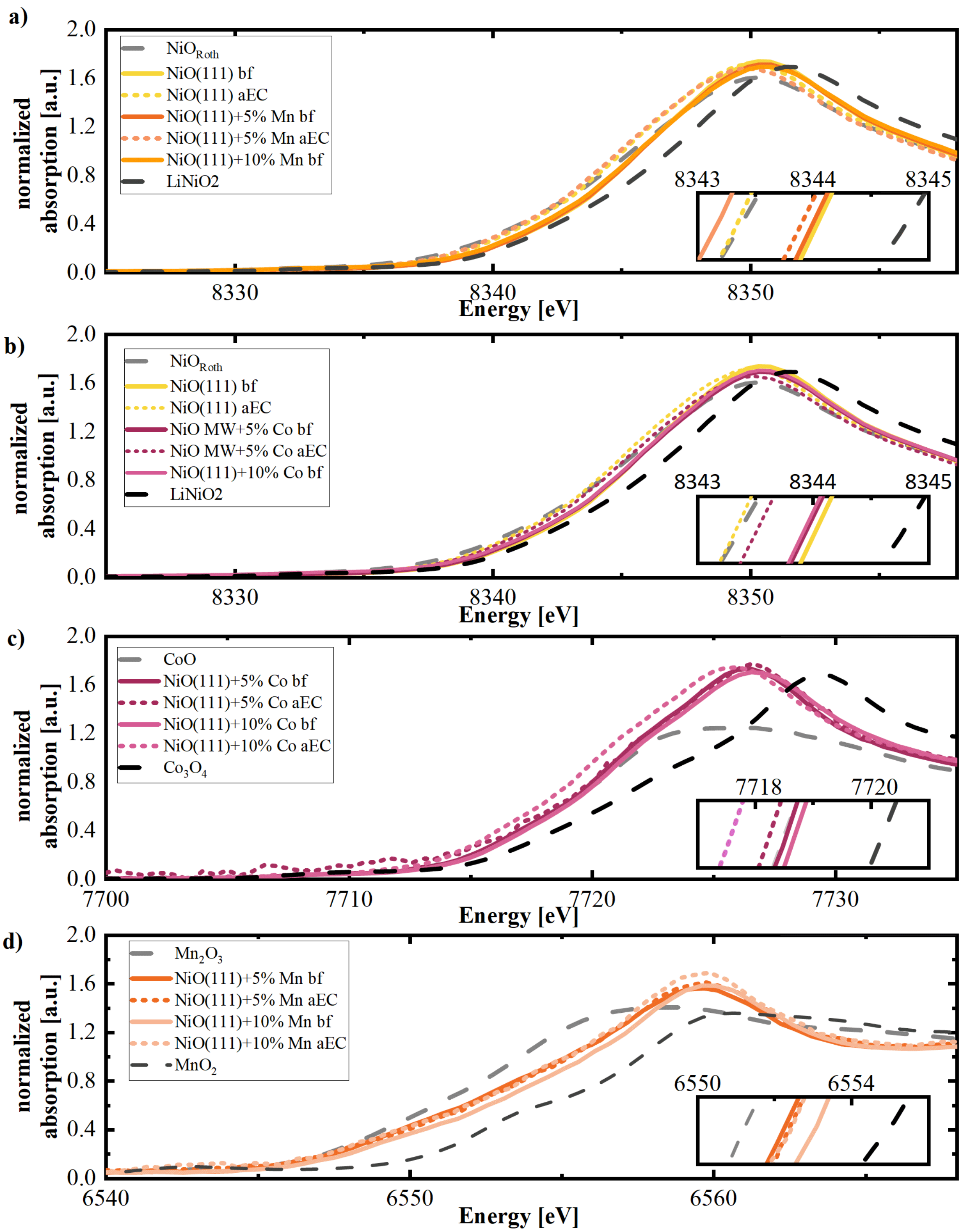

Figure S20: XANES data of the samples for a) the Ni k edge, b) the Mn k edge and c) the Co k edge as pristine sample and after electrochemical treatment aEC.

Table S7: Ni, Co and Mn k edge energies from the integral under the normalized XANES of the samples including the reference materials.

**Sample**	**Ni k edge [eV]**
$Ni^{II}O_{Roth}$	8342.3
$LiNi^{III}O2$	8343.8
NiO(111)	8342.9
NiO(111) aEC	8342.3
NiO(111)+5%Co	8342.7
NiO(111)+10%Co	8342.7
NiO(111)+5%Co aEC	8342.3
NiO(111)+10%Co aEC	8341.9
NiO(111)+5%Mn	8342.8
NiO(111)+10%Mn	8342.7
NiO(111)+5%Mn aEC	8341.9
NiO(111)+10%Mn aEC	8342.3

**Sample**	**Mn k edge [eV]**
$Co^{II}O$	7718.59
$Co^{II/III}{}_3O_4$	7720.34
NiO(111)+5%Co	7718.47
NiO(111)+10%Co	7718.20
NiO(111)+5%Co aEC	7718.67
NiO(111)+10%Co aEC	7717.58

**Sample**	**Co k edge [eV]**
Mn_2O_3	6550.91
MnO_2	6554.55
NiO(111)+5%Mn	6551.82
NiO(111)+10%Mn	6552.57
NiO(111)+10%Mn aEC	6551.88
NiO(111)+5%Mn aEC	6551.99

S16. EXAFS of different Mn k edge reference Materials

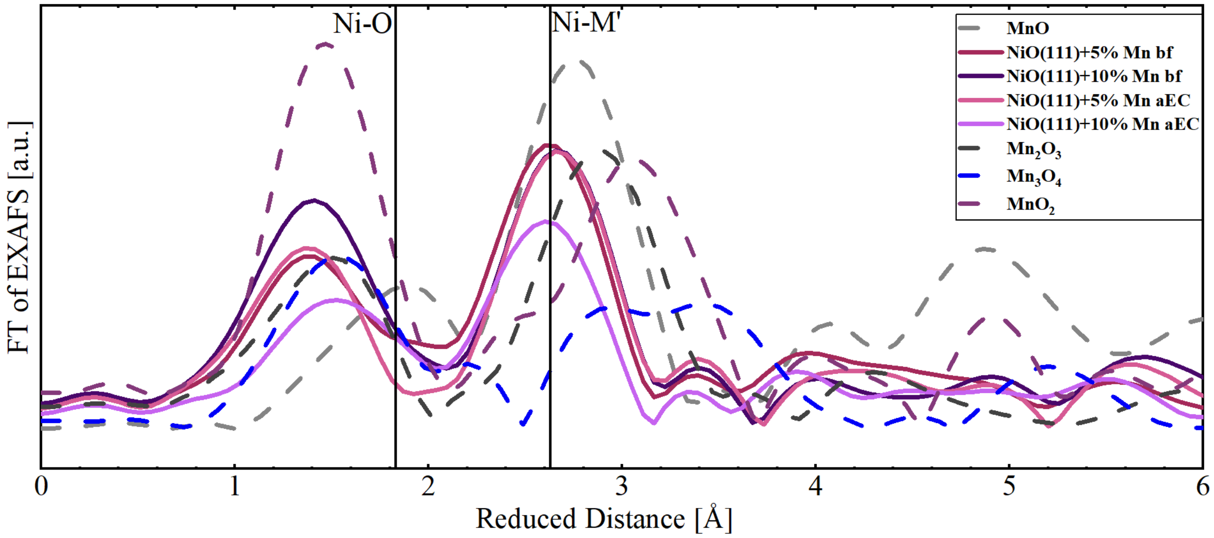

Figure S 21: Fourier transformation of the Mn-K edge EXAFS for the prepared samples and reference materials. The reduced distance between Ni-M' as well as the Ni-O from the Ni-K edge are marked as a guide to the eye.

S17. XPS Survey spectra

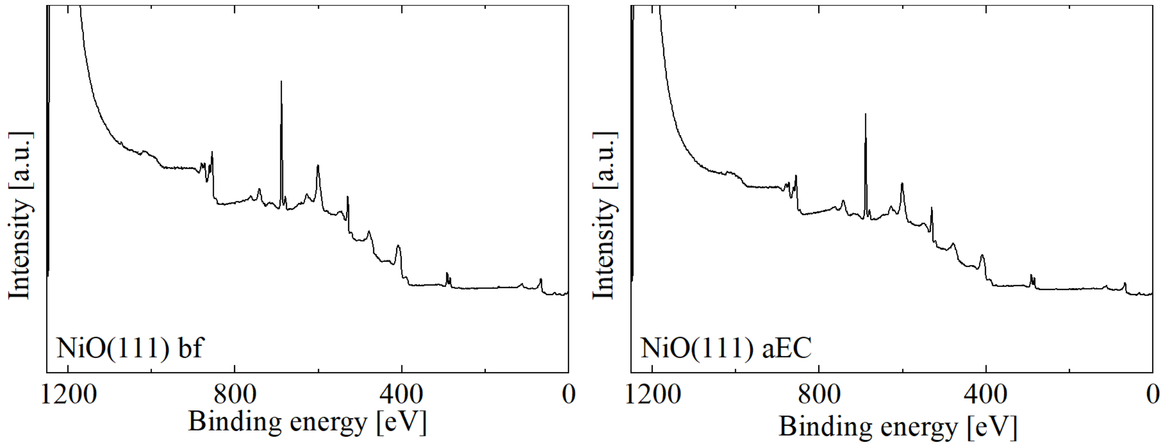

Figure S22: Survey XP spectra of NiO(111) before (bf) and after electrochemical treatment (aEC).

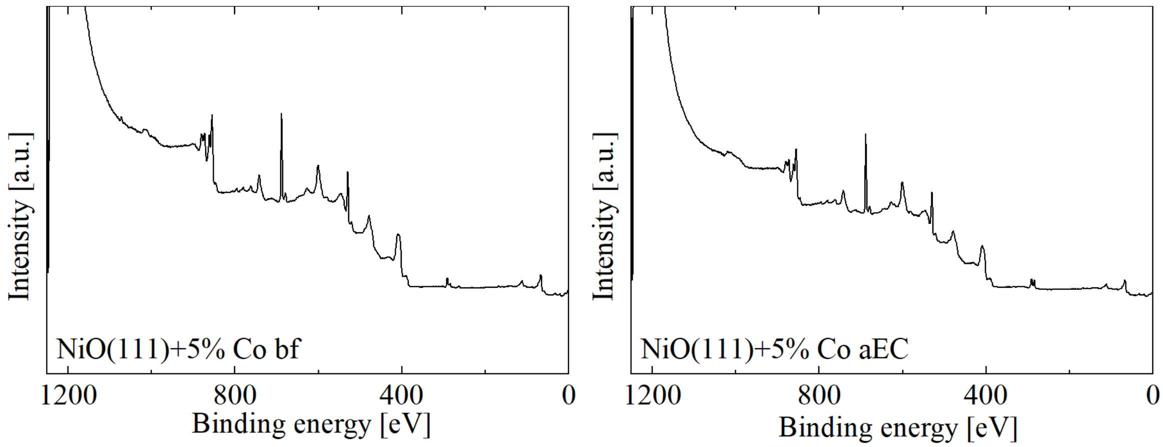

Figure S23: Survey XP spectra of NiO(111)+5% Co before (bf) and after electrochemical treatment (aEC).

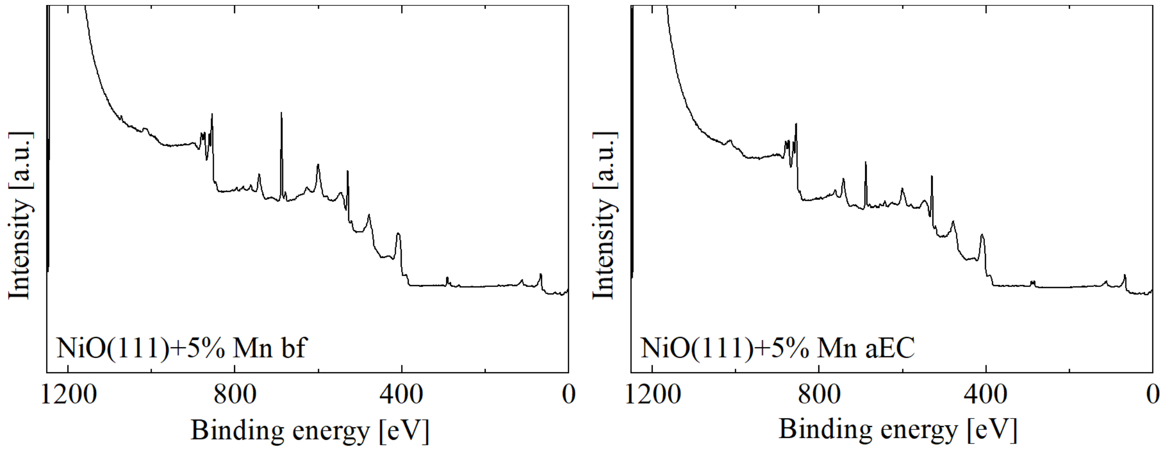

Figure S24: Survey XP spectra of NiO(111)+5% Mn before (bf) and after electrochemical treatment (aEC).

S18. XPS Co and Mn 2p spectra

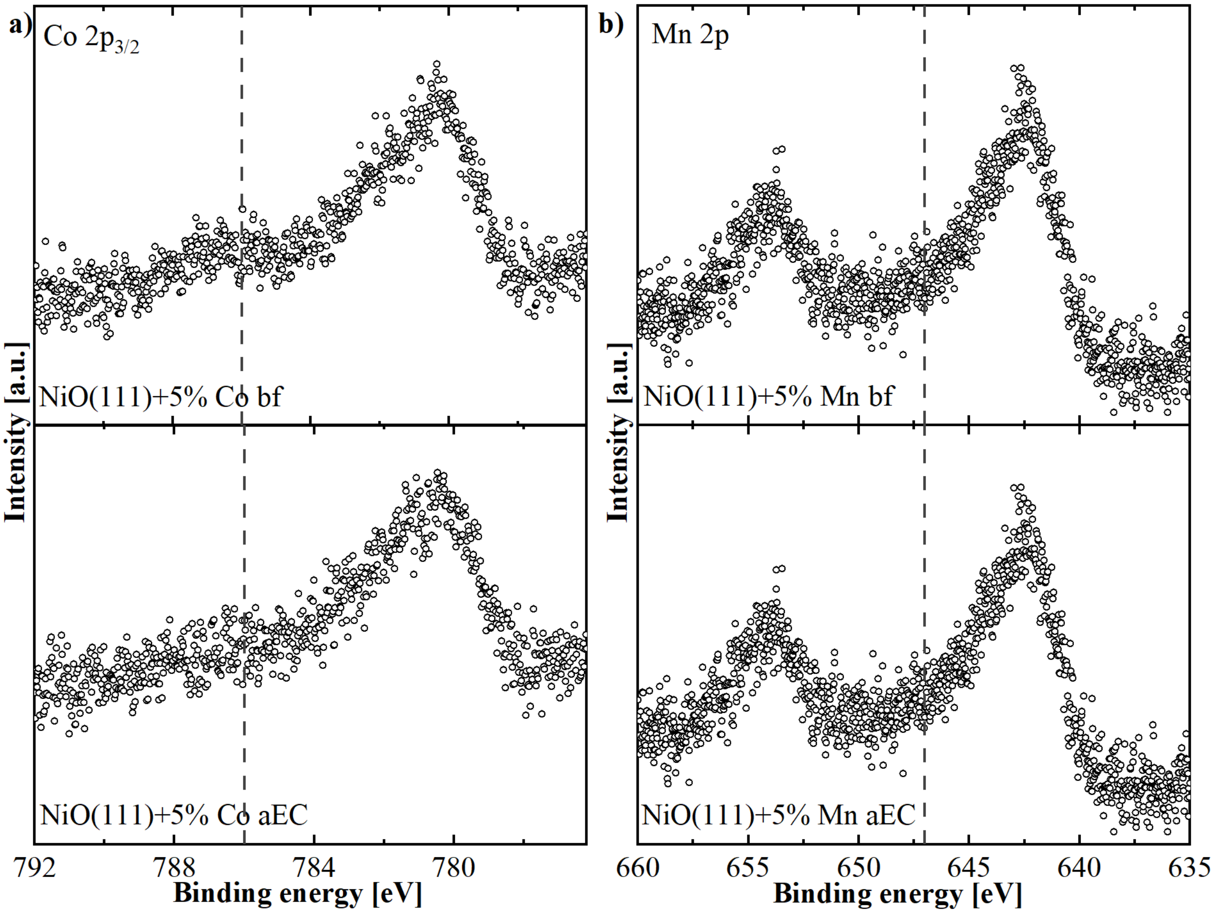

Figure S25: a) Co $2p_{3/2}$ spectra of NiO(111)+ 5% Co with a mark for the prominent satellite at 786 eV as well as b)Mn 2p spectra of the 5% Mn sample with a mark for the prominent satellite at 647 eV, both before (bf) and after electrochemical treatment (aEC).

S19. XPS C 1s spectra

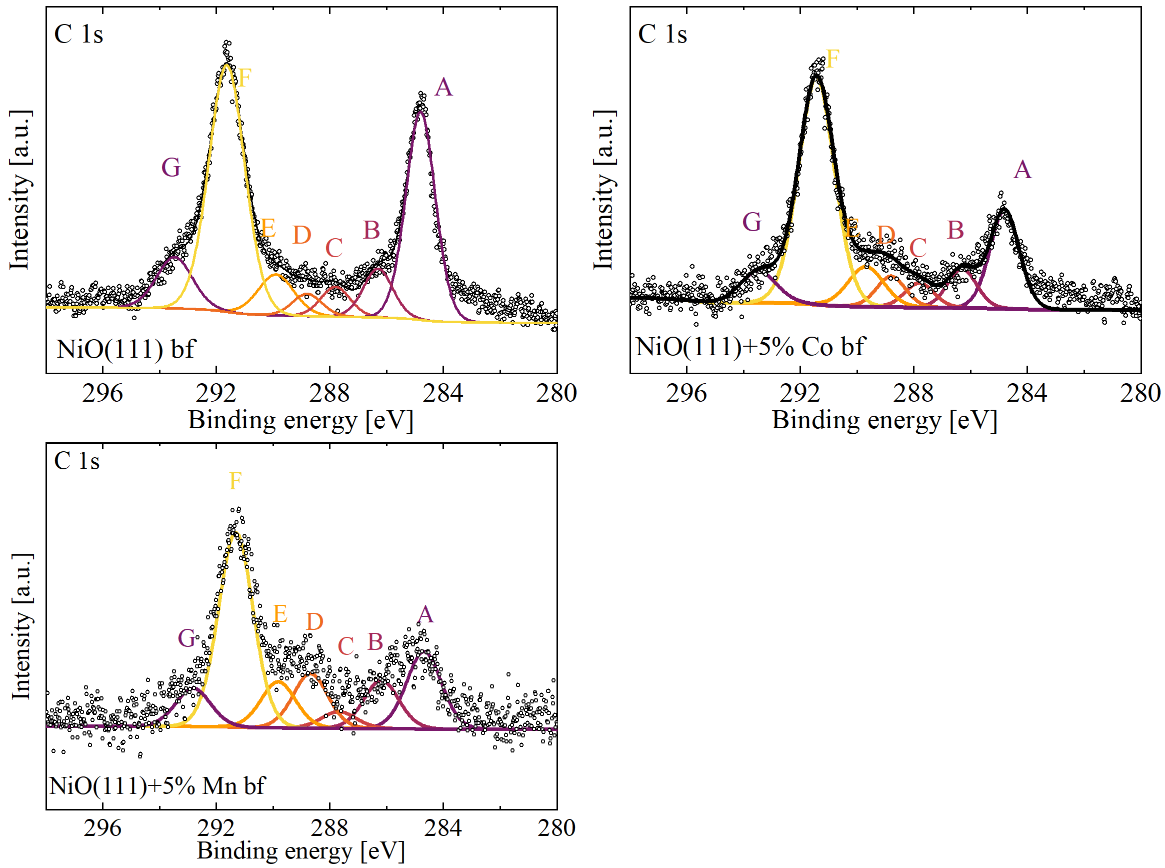

Figure S 26: C 1s XP spectra of NiO(111) as well as the 5% Co and the 5% Mn sample before (bf) electrochemical treatment. The feature A was used for calibration to 248.8 eV.

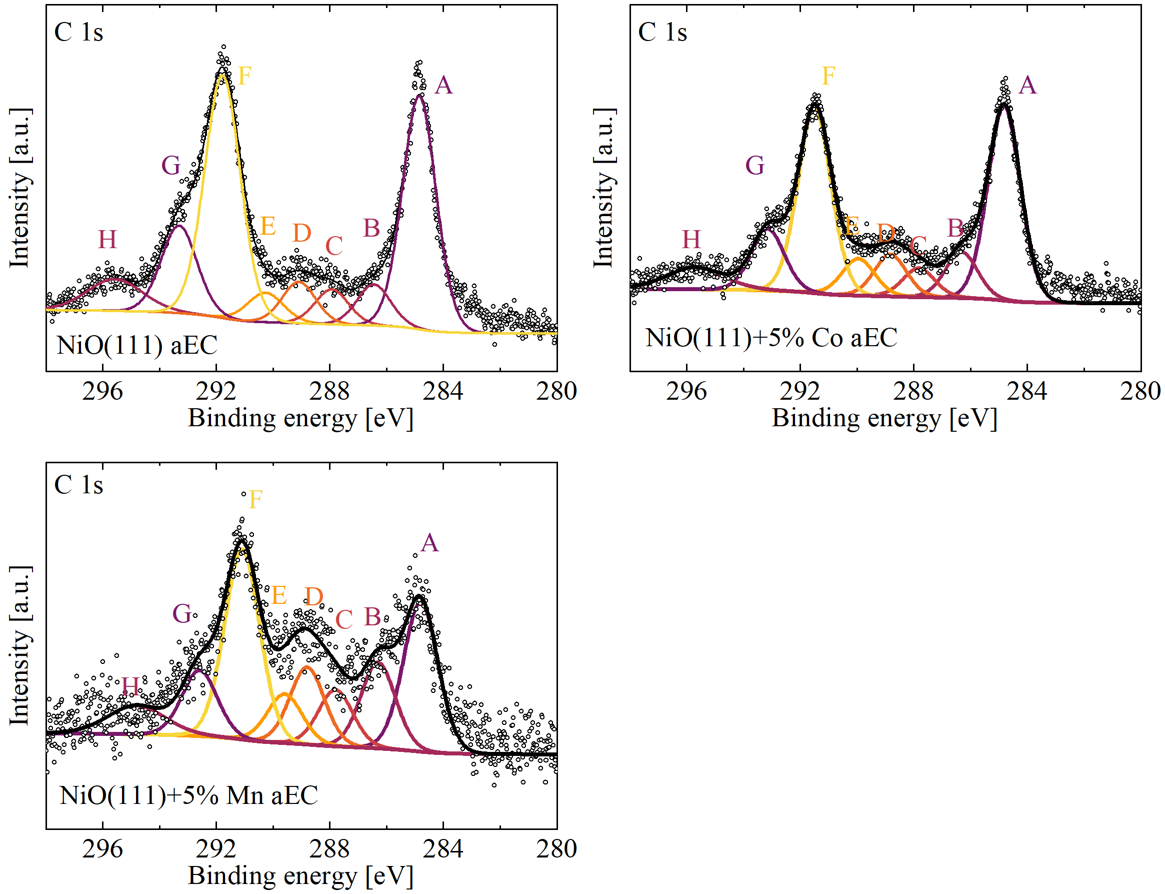

Figure S 27: C 1s XP spectra of NiO(111) as well as the 5% Co and the 5% Mn sample after electrochemical treatment (aEC). The feature A was used for calibration to 248.8 eV.

References

(1) Bisen, O. Y.; Baumung, M.; Tatzel, M.; Volkert, C. A.; Risch, M. Manganese dissolution in alkaline medium with and without concurrent oxygen evolution in LiMn2O4. *Energy Adv.* **2024**, *3* (2), 504–514. DOI: 10.1039/D3YA00434A.
(2) Vos, J. G.; Koper, M. Examination and prevention of ring collection failure during gas-evolving reactions on a rotating ring-disk electrode. *J. Electroanal. Chem.* **2019**, *850*, 113363. DOI: 10.1016/j.jelechem.2019.113363.
